# Genetic mapping of centromeres in the nine *Citrus clementina* chromosomes using half-tetrad analysis and recombination patterns in unreduced and haploid gametes

**DOI:** 10.1186/s12870-015-0464-y

**Published:** 2015-03-08

**Authors:** Pablo Aleza, José Cuenca, María Hernández, José Juárez, Luis Navarro, Patrick Ollitrault

**Affiliations:** Centro de Protección Vegetal y Biotecnología, Instituto Valenciano de Investigaciones Agrarias (IVIA), Moncada, Valencia Spain; CIRAD, UMR AGAP, Avenue Agropolis - TA A-75/02 F‐34398, Montpellier, France

**Keywords:** Clementine, Triploid, Second-division restitution, Chromosome interference, Physical location, Genetic recombination

## Abstract

**Background:**

Mapping centromere locations in plant species provides essential information for the analysis of genetic structures and population dynamics. The centromere’s position affects the distribution of crossovers along a chromosome and the parental heterozygosity restitution by 2n gametes is a direct function of the genetic distance to the centromere. Sexual polyploidisation is relatively frequent in *Citrus* species and is widely used to develop new seedless triploid cultivars. The study’s objectives were to (i) map the positions of the centromeres of the nine *Citrus clementina* chromosomes; (ii) analyse the crossover interference in unreduced gametes; and (iii) establish the pattern of genetic recombination in haploid clementine gametes along each chromosome and its relationship with the centromere location and distribution of genic sequences.

**Results:**

Triploid progenies were derived from unreduced megagametophytes produced by second-division restitution. Centromere positions were mapped genetically for all linkage groups using half-tetrad analysis. Inference of the physical locations of centromeres revealed one acrocentric, four metacentric and four submetacentric chromosomes. Crossover interference was observed in unreduced gametes, with variation seen between chromosome arms. For haploid gametes, a strong decrease in the recombination rate occurred in centromeric and pericentromeric regions, which contained a low density of genic sequences. In chromosomes VIII and IX, these low recombination rates extended beyond the pericentromeric regions. The genomic region corresponding to a genetic distance < 5cM from a centromere represented 47% of the genome and 23% of the genic sequences.

**Conclusions:**

The centromere positions of the nine citrus chromosomes were genetically mapped. Their physical locations, inferred from the genetic ones, were consistent with the sequence constitution and recombination pattern along each chromosome. However, regions with low recombination rates extended beyond the pericentromeric regions of some chromosomes into areas richer in genic sequences. The persistence of strong linkage disequilibrium between large numbers of genes promotes the stability of epistatic interactions and multilocus-controlled traits over successive generations but also maintains multi-trait associations. Identification of the centromere positions will allow the development of simple methods to analyse unreduced gamete formation mechanisms in a large range of genotypes and further modelling of genetic inheritance in sexual polyploidisation breeding schemes.

**Electronic supplementary material:**

The online version of this article (doi:10.1186/s12870-015-0464-y) contains supplementary material, which is available to authorized users.

## Background

The centromere is a specialised structure within a chromosome, recognisable morphologically as the primary constriction. Centromeres mediate chromosome segregation at mitosis and meiosis, provide the proteinaceous kinetochore, promote sister chromatid cohesion and suppress recombination. Centromeric regions include large arrays of satellite DNA flanked by middle repetitive DNA rich in repetitive elements including transposons, retroelements and pseudogenes [1]. Although the structural characteristics of plant centromeres have been well defined, there is no conservation of centromeric sequences and these differ both from chromosome to chromosome and between species [1-3], highlighting the rapid rate of centromere evolution [1,3-5]. Centromere mapping allows the development of improved linkage maps, deciphering of chromosome arms, investigation of crossover events and understanding of crossover interference during meiosis. It is thus essential for analysis of the genetic structures of animal and plant species [6-9].

In some plant species, which keep their meiotic products together in tetrads, centromere mapping can be performed via tetrad analysis [10]. However, this mechanism is limited to a few species [9,10]. In many more species, the centromeres can be localised using half-tetrad analysis (HTA) of unreduced (2n) gametes. Unreduced gametes are the main cause of polyploidisation in plant species [11-14]. Their presence has been described in several crop species, including alfalfa [15], maize [16], *Solanum* [9,17-20] and *Citrus* species [21,22]. Several meiotic aberrations related to spindle formation, spindle function and cytokinesis result in 2n gamete formation in plants. The type of 2n gamete produced, however, depends essentially upon which of two basic processes, first-division restitution (FDR) and second-division restitution (SDR), has occurred. FDR and SDR depend upon the mode of nuclear restitution [12,15] and result from the omission of the first or the second meiotic division, respectively. A FDR 2n gamete contains non-sister chromatids, whereas an SDR 2n gamete contains two sister chromatids [23]. This implies that the patterns of parental heterozygosity restitution (HR) seen along the chromosome with respect to the genetic distance from the centromere will be completely opposite in the two types of diploid gamete [9,15,17,24]. Molecular marker analysis is therefore a powerful means of identifying the mechanism underlying unreduced gamete formation [15,25-27] and of locating centromeres genetically [6,9,17,24,28,29]. Tavoletti *et al*. [15] developed a multilocus maximum likelihood method of HTA assuming complete chromosome interference. Cuenca *et al*. [24] proposed an alternative approach based on HR functions along a chromosome and their relationship to locus-centromere genetic distance, allowing different chromosome interference models to locate the positions of the centromeres in linkage groups (LGs).

Citrus species make up the world’s leading fruit crop with 131.3 million tons produced in 2012 [30]. International efforts have led to an increase in genomic and genetic resources, allowing better preservation of biodiversity and improvements in breeding strategies and their efficiency. Ollitrault *et al.* [31] established the current reference clementine genetic map (*Citrus clementina* Hort. ex Tan). This includes 961 co-dominant markers spread across nine LGs and spanning 1084.1 cM, with an average marker spacing of 1.13 cM. Recently, a reference whole clementine genome sequence, anchored on the clementine genetic map, was obtained by the International Citrus Genome Consortium [32] from a haploid plant of ‘Clemenules’ clementine [33].

Diploidy is the general rule in *Citrus* and related genera, and the basic chromosome number is × = 9. However, sexual polyploidisation is relatively frequent and is central to the current approach of developing triploid citrus-breeding programmes with an aim of producing new seedless mandarin cultivars [34,35]. Esen and Soost [21] proposed that, in *Citrus*, unreduced ovules arose from the abortion of the second meiotic division. This hypothesis was corroborated by molecular marker analyses of clementine [26] and ‘Fortune’ mandarin (*C. clementina* × *C. tangerina*) [24]. However, Chen *et al*. [36] proposed that female 2n gametes produced by the sweet orange (*C. sinensis* (L.) Osb.) were generated by FDR. This paved the way for HTA mapping of centromere locations in *Citrus*, although only the centromere of LG 2 has been mapped to date [24].

The major objective of this work was to establish the genetic location of centromeres within the nine LGs of the clementine genetic map [31] anchored on the sequences of the corresponding nine chromosome of the citrus haploid set [32]. Centromere positions were located using HTA by determining the genotypes of 87 triploid hybrids, recovered from 2× × 2× sexual hybridisation, at 104 co-dominant molecular markers, including simple sequence repeats (SSRs), insertion-deletions (InDels) and single nucleotide polymorphisms (SNPs). This information was used to determine the distributions of crossovers and reveal the variation in interference levels across the different chromosome arms in clementine 2n gametes. Finally, we analysed the pattern of genetic recombination along the physical sequences of haploid gametes with respect to the centromere location and frequency of genic sequences.

## Methods

### Plant material

The mechanism of 2n gamete formation was investigated in the progeny of 87 triploid hybrids recovered from a cross between diploid ‘Fina’ clementine (female parent) and ‘Nadorcott’ tangor (male parent). Practical details of recovery of the triploid hybrids from 2× × 2× hybridisation, using embryo rescue followed by flow cytometry to select triploid hybrids, may be found in Aleza *et al.* [35]. No selection was made between the triploid hybrids and all genotypes were grown at the Instituto Valenciano de Investigaciones Agrarias (IVIA, Moncada, Valencia, Spain). Genomic DNA was isolated from triploid hybrids and their parents using the Plant DNAeasy kit (Qiagen, Madrid, Spain), following the manufacturer’s protocol.

### Genotyping of triploid progeny using molecular marker analysis

The male and female parents and 87 triploid hybrids were genotyped using a total of 104 molecular markers (48 SSRs, 11 InDels and 45 SNPs). Genotyping revealed heterozygosity of the ‘Fina’ clementine female parent and polymorphism within the ‘Nadorcott’ tangor male parent. SNP markers were selected using previous genotyping data obtained from the Illumina Golden Gate™ platform [37]. These markers are widely distributed across the current genetic map of Clementine [31].

PCR amplifications of genomic DNA with the 59 SSR and InDel markers were performed using a Thermocyclerep gradient S (Eppendorf®). Each reaction contained 0.8 U Taq DNA polymerase (Fermentas®), 2 ng/mL *Citrus* DNA, 0.2 mM wellRED (Sigma®) dye-labelled forward primer, 0.2 mM non-dye-labelled reverse primer, 0.2 mM each dNTP, 10× PCR buffer and 1.5 mMMgCl_2_in a final volume of 10 mL. The PCR protocol was as follows: an initial denaturation at 94°C for five minutes followed by 40 cycles of 30 seconds at 94°C, one minute at 50°C or 55°C, 45 seconds at 72°C, and a final elongation step of four minutes at 72°C. Capillary electrophoresis was performed using a CEQ™ 8000 Genetic Analysis System (Beckman Coulter Inc., Fullerton, CA, USA). Data were collected and analysed using GenomeLab® GeXP (Beckman Coulter Inc.) version 10.0 software. Allele dosage was calculated using the MAC-PR (microsatellite DNA allele counting-peak ratio) method [38], validated in *Citrus* by Cuenca *et al*. [24].

The genotypes of triploid progenies at 45 SNP markers were determined using KASPar technology by Kbioscience® services (now LGC Genomics; http://www.lgcgenomics.com). The KASPar™ Genotyping System is a competitive, allele-specific dual Förster Resonance Energy Transfer (FRET) based assay for SNP genotyping. Primers were designed by LGC Genomics based on the SNP locus flanking sequence (approximately 50 nucleotides each side of the SNP). A detailed explanation of the specific conditions and reactions may be found in Cuppen [39]. Allele doses in the heterozygous triploid hybrids were determined using the relative allele signals of the SNP markers, based on competitive allele-specific PCR, as described by Cuenca *et al.* [40]. Detailed information on all the markers used in this study is given in Additional file 1.

### Identification of the parent producing the unreduced gamete and inference of the unreduced gamete genotype

The use of markers which differentiated between the alleles from the female and male parents allowed the unequivocal identification of the parent which produced the 2n gamete for each triploid hybrid, based on HR or allele dosage estimation. Once the origin of the 2n gamete had been identified, the allelic configurations of the unreduced gametes were inferred using the data obtained from genotyping the triploid hybrids, as previously described in Cuenca *et al.* [24].

The origin of the 2n gamete for each hybrid was determined by identifying which parent had passed on a double dose of genetic information. For markers scored in the parents (female × male) as A_1_A_2_ × A_1_A_1_ or A_1_A_2_ × A_1_A_3_, identification of the triploid hybrids as A_1_A_2_A_2_ or A_2_A_2_A_3_ (i.e., possessing a double dosage of A_2_, the allele specific to the female parent) revealed the 2n gamete had originated from the female parent but the observation of A_1_A_3_A_3_ or A_2_A_3_A_3_ (i.e., with a double dosage of A_3_, the allele specific to the male parent) indicated a male origin for the 2n gamete. For markers scored as A_1_A_2_ × A_3_A_3_ in the parents, the triploid hybrids A_1_A_2_A_3_, A_1_A_1_A_3_ or A_2_A_2_A_3_ indicated a maternal origin for the unreduced gamete, whilst A_1_A_3_A_3_ or A_2_A_3_A_3_ indicated a paternal origin. For markers scored as A_1_A_2_ × A_3_A_4_ in the parents, the triploid hybrids A_1_A_1_A_3_, A_1_A_1_A_4_, A_1_A_2_A_3_, A_1_A_2_A_4_, A_2_A_2_A_3_ and A_2_A_2_A_4_ indicated a female origin for the 2n gamete and the hybrids A_1_A_3_A_3_, A_2_A_3_A_3_, A_1_A_3_A_4_, A_2_A_3_A_4_, A_1_A_4_A_4_ and A_2_A_4_A_4_ a male origin.

Once the parent producing the 2n gamete had been identified, the allelic configurations of the unreduced gametes were inferred from triploid hybrid genotyping, as previously described by Cuenca *et al.* [24]. For loci where the parental alleles were completely different (for example, A_1_A_2_ × A_3_A_4_), the genotype of the 2n gamete was directly read from the triploid hybrid structure. If both parents shared one allele (for example, A_1_A_2_ × A_2_A_2_ or A_1_A_2_ × A_2_A_3_), the inference of the 2n female gamete structure was carried out using the estimated allele dosage for those triploid hybrids that inherited the common allele from the male parent. For each locus, the parental heterozygosity restitution (HR) was calculated as the percentage of individuals with the same heterozygous allelic configuration as that of the female parent.

### Identification of the mechanism of unreduced gamete formation by single-locus analysis

The parental HR for 2n gametes arising from FDR or SDR directly relates to the genetic distance of a given locus from the centromere, but the two types of diploid gamete produce a completely opposite pattern of HR [24]. In the absence of crossovers, all loci heterozygous in the parent will be heterozygous in FDR gametes but, in SDR gametes, all the loci situated between the centromere and the first crossover will be homozygous. As the genetic distance from the centromere increases, HR decreases in the case of FDR; it remains, however, at over 50% regardless of the chromosome interference model. In the case of SDR, HR increases with the genetic distance until it reaches 100% under the total chromosome interference model [9,15,17,24]. In this study, the identification of mechanism was based on an analysis of HR at each locus across the entire inferred 2n gamete population. In the absence of previous knowledge of the relative positions of markers to the centromere, the observation that HR is greater than 50% at a single locus is not informative, since it could result from either FDR or SDR. Theoretical HR values below 50%, however, are only found in the case of SDR [9]. When such low values of HR were observed at a marker, a LOD score test was calculated to compare the probabilities of the observed level of HR occurring, under both the SDR and FDR hypotheses. In the case of SDR, the highest probability of the observed HR occurs at the centromere position, leading to a match between the theoretical and observed proportions of heterozygous gametes. In the case of FDR, the best fit between the theoretical proportion of heterozygous gametes and observed data is obtained for a theoretical proportion of 50%, because 50% is the minimum value of HR expected under the hypothesis of FDR. Thus, logarithm of the odds ratios (LOD) were estimated as follows:

$$ \mathrm{L}\mathrm{O}\mathrm{D}={ \log}_{10}\left[\frac{p(SDR)}{p(FDR)}\right]={ \log}_{10}\left[\frac{h^{nh}\cdotp {\left(1-h\right)}^{\left(1-h\right)\cdotp n}}{0.5^{nh}\cdotp {(0.5)}^{\left(1-h\right)\cdotp n}}\right] $$

With h being the HR observed for the marker and n the number of genotypes analysed at this marker. LOD = 3 (*i.e*., the probability of the SDR hypothesis being more than 1000-fold that of FDR) was considered the significance threshold for concluding in favour of SDR rather than FDR.

### Identifying the preliminary locations of centromeres using HR functions under no interference and partial interference chromosome models

The methodology of Cuenca *et al.* [24] was used to identify the preliminary locations of the centromeres. This method is based on comparing the observed HR values along each LG with the theoretical restitution functions under the SDR mechanism of 2n gamete formation for both the no interference and partial interference models on a chromosome arm (Cx(Co)^4^). These functions were derived from those developed by Zhao and Speed [41] for ordered tetrads, based on the random spindle–centromere attachment hypothesis [42], and extended by the same authors to HTA [43]. Discrepancies between the different models (no interference and partial interference coupled with the different location of the centromere) and the observed data were estimated by the sum of the squared differences between the observed and theoretical values at the marker map positions. Let Fit(c) be the value of the sum of the squared distance for each position of the centromere for one interference model; the best theoretical centromere position under this model is deduced by searching c, which minimises Fit(c). The confidence interval (95%) for the centromere position was estimated by bootstrap on the loci (500 bootstraps).

The locations of genetic markers were obtained from the reference clementine map [31] established using Kosambi’s map function and, for the no interference model, the genetic positions were established from the same genotypic data [31] but using Haldane’s map function.

### Centromere mapping using multilocus half-tetrad structure analysis

Multilocus analyses were performed on the 87 hybrid triploid genotypes at four loci in each LG. These four loci were selected following the preliminary localisation of the centromeres, as described above, and consisted of two flanking loci on each side of the preliminary location. The analyses were conducted according to Tavoletti *et al*. [15] and assumed multiple crossovers did not occur between contiguous markers. The position of the centromere was moved virtually stepwise at intervals of 0.01 cM along the LG from before the first selected marker (M1) to after the last selected marker (M4), and the probability of the observed populations occurring was estimated at each position. The best estimate for the centromere location was that producing the highest probability. The confidence interval was calculated using the LOD drop-off method [44].

### Crossover interference analysis

After determination of the centromere position, three-point linkage mapping was used to estimate the level of crossover interference for each chromosome arm. The centromere (considered to be homozygous) was used as the first point and two markers were selected in each arm. The chromosome interference coefficient (IC) was defined as follows by Griffiths *et al.* [42]:

$$ \mathrm{I}\mathrm{C}=1-\left[\frac{rd}{r_{C{M}_1}\cdotp {r}_{M_1{M}_2}}\right] $$

When r_CM1_ denotes the observed recombination rate (heterozygous to homozygous and *vice versa*) between the centromere and locus 1, r_M1M2_ is the observed recombination rate between locus 1 and 2 and rd is the observed rate of double recombination between the centromere and locus 2.

### Relationship between genetic and physical locations

The physical locations of the genetic markers used to establish the clementine genetic map were identified by searches, using the flanking sequences of the markers [31,38], of the clementine reference genome [32] using the blast N option [45] of the online Galaxy tool (http://gohelle.cirad.fr/galaxy/).

The cM/Mb rates between genetic and physical positions of markers for each LG were estimated using local linear regression. Each LG was divided in intervals of two or three Mb and the slope between the values of the genetic and physical marker positions was calculated for each interval.

## Results and discussion

### Mechanism of 2n gamete formation

#### Parental origin of the 2n gamete producing triploid hybrids in 2x × 2x crosses

Of the 104 markers analysed, six loci (mCrCIR01C06, mCrCIR07B05, Cx6F03, CID0591, CID2493 and MEST473) were able to differentiate completely between the female and male parents and thus enabled unequivocal identification of the parent producing the 2n gamete for each triploid hybrid (Additional file 1). The diploid gamete was of maternal origin in all the triploids analysed and it was therefore possible to infer the 2n gamete structure of each hybrid. Our results were in agreement with previous, pioneering works, which concluded citrus triploid hybrids arose from 2n megagametophytes [21,46,47]. Cytogenetic studies [48,49] showed triploid embryos are associated with pentaploid endosperm, a strong indication that they result from the fertilisation of unreduced ovules by normal haploid pollen [48]. Depending on the genotype, the frequency of duplication among female gametes varied from between less than 1% to more than 20% [50].

#### Mechanism of 2n megagametophyte formation in Clementine

Potential allelic segregation distortion in the 2n gamete population was tested at each marker using χ^2^-analysis (0.05 probability threshold) with the Bonferroni correction for multiple testing applied (Additional file 2). None of the 104 markers tested displayed significant segregation distortion.

**Figure 1:**
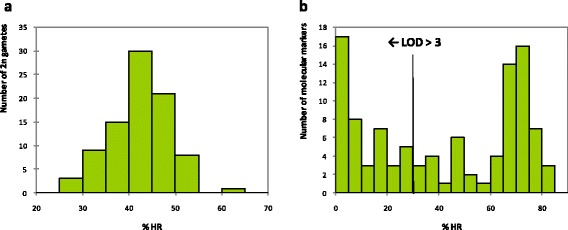
**Heterozygosity restitution percentages (%HR) for all 2n gametes and molecular markers used in this study. a**: Distribution of %HR estimated for each 2n gamete. **b**: Distribution of %HR estimated for each molecular marker and %HR corresponding to LOD scores greater than three with significance for SDR mechanism of 2n gamete formation.

Maternal HR in each 2n gamete varied between 27.18 and 61.39% across the loci analysed, with a mean value of 42.45%. The unimodal distribution of HR in the 2n megaspores suggested all the 2n gametes arose from the same mechanism (Figure 1a). Analyses of 2n gamete origin and the centromere location were conducted under this hypothesis and it was confirmed *a posteriori* by a LOD score analysis conducted at the individual level (Additional file 3).

The rate of HR at a population level was calculated for each of the 104 loci (Figure 1b). The average rate across all loci was 41.73% but values ranged from 0% for the marker CiC6278-01, to 82.76% for the marker mCrCIR03G05. HR values were lower than 50% at 57 of the markers analysed. LODs of SDR/FDR probabilities were calculated for these markers, and found to vary between 0 and 23.8 (Additional file 2). When a LOD = 3 was taken as the significance threshold for accepting SDR rather than FDR, the corresponding%HR threshold was 30.35%. Forty-two markers displayed an LOD > 3 (*i.e.*,%HR < 30.35%; Figure 1b), supporting the SDR hypothesis and ruling out the possibility of FDR in this population.

These results are consistent with earlier studies which indicated an SDR origin for 2n megagametophytes in clementine [24,26] and ‘Fortune’ mandarin [51]. FDR, however, may be the mechanism producing unreduced female gametes in sweet orange [36] and in lemon [51], although these conclusions were based on HR values greater than 50% in a small number of markers without any knowledge of the distances between markers and centromeres. They are therefore questionable, since such values could also result from SDR if there was a large distance between the markers analysed and the centromeres. In the absence of additional information on centromere position, drawing a definitive conclusion of FDR would require the analysis of a considerable number of genotypes with a large number of markers well distributed across the different chromosomes.

SDR is frequently observed in plants in the formation of 2n ovules whereas, even within the same species, 2n pollen may be produced by both FDR and SDR [12]. The mechanism of 2n gamete formation is a strong determinant of the genetic and phenotypic structures of polyploid hybrid populations, because of its effect on parental HR. Knowledge of the particular meiotic nuclear restitution mechanism producing the unreduced gametes is crucial, therefore, for the optimisation of plant breeding strategies based on sexual hybridisation [52,53]. Several studies of genetic markers indicate that gametes formed by FDR transmit 70–80% of parental heterozygosity to progeny but those gametes formed by SDR transmit only 30–40% [54-56]. These values are in agreement with our estimation of 41.7% transmission in clementine.

In progenies produced by 2n gametes derived from FDR, parental heterozygosity and epistatic interactions are maintained across a higher number of individuals and loci than in SDR-derived progenies. Moreover, because a greater percentage of the parental genome is transferred intact following FDR than SDR, FDR produces a more uniform population of 2n gametes and thus a lower range of variation between individuals is expected in populations derived from FDR [57]. For this reason, gametes derived from FDR are considered better than those from SDR for breeding purposes, as they create offspring similar to the female parent and thus allow transmission of the genetic gain at the maternal level into progeny and optimisation of heterotic responses [17]. The superiority of progeny resulting from sexual polyploidisation involving 2n gametes produced by FDR has been demonstrated, for instance, in potato [58,59]. In contrast, 2n gametes generated by SDR produce more variable offspring and thus create a greater number of new genetic combinations, increasing the likelihood of obtaining novel phenotypes [60].

For specific characters controlled by a major gene, the allelic effect (dominance; recessivity; heterosis) and genetic distance to the centromere are both crucial in determining the proportion of the progeny that will show the favourable trait. With respect to tuber yield in potato, for example, increased yield due to heterosis in FDR-derived progeny is associated with the location of genes with major effects on tuber yield between the centromeres and proximal crossovers [61]. In *Citrus*, the recessive resistance gene for Alternaria Brown Spot fungus disease is located close to the centromere. This situation favours the operation of SDR and allows for up to 40% of the progeny showing disease resistance when the heterozygous ‘Fortune’ mandarin is used as the female parent [62]. Therefore, being able to locate the position of centromeres accurately on a genetic map anchored on the annotated whole genome sequence is a critical step for further modelling of the inheritance of traits in breeding schemes utilising sexual polyploidisation.

### Centromere mapping

Centromere mapping by HTA is used in plants and animals to integrate centromeres with linkage maps [6,9,15,24,28,62]. In this study, mapping of the centromere positions was done in two steps: initially, we identified a preliminary position for each centromere by comparing the observed and theoretical patterns of HR along the LG under the SDR mechanism of female 2n gamete formation and the no interference and partial chromosome interference models, according to the methodology of Cuenca *et al.* [24]. Once the preliminary location of the centromere had been established, we selected markers flanking this position and used multilocus HTA to locate the centromeres more accurately [15].

#### Preliminary location of centromeres

**Figure 2:**
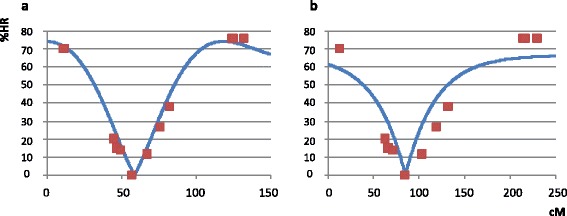
**Observed heterozygosity restitution percentages (%HR) for markers on LG 2 (squares) and theoretical HR (line) for the best-fitting centromere position. a**: under SDR and the Cx(Co)^4^ model of partial interference (Kosambi’s map function) and **b**: under the SDR model without crossover interference (Haldane’s map function). Markers on the x-axis are shown according to their position on the clementine genetic map [31].

Between nine and fourteen molecular markers per LG were used to locate the position of the centromere by comparing the observed and theoretical patterns of HR rate within each LG. Earlier results had allowed us to discard the FDR mechanism and so we only tested the two interference models under the SDR hypothesis; Figure 2 displays the pattern of HR for each model of interference on LG 2. As we moved from one end of LG 2 to the other, HR decreased from 70.11% at the SSR marker mCrCIR02D09 to 0% at the SNP marker CiC6278-01, and then increased again to 75.86% at the SSR marker JK-TAA41. A better adjustment between the theoretical curves and the observed pattern of HR was obtained using the partial interference model.

The patterns of HR observed across all LGs provided a picture of a typical 2n gamete population resulting from SDR (Additional file 4). For each LG, the best fit value (the lowest value of the sum of the squared distance between theoretical and observed HR for each marker for the best identified location of the centromere) was obtained using the partial interference model. The ‘no interference/partial interference’ ratios ranged between 1.4 for LG 8 and 8.5 for LG 7 (Table 1). The maximum value of theoretical HR for the no interference model was 66.67% [63,64] and 38 markers displayed a HR value greater than 66.67%. The statistical significance was tested using χ^2^-analysis and seven markers displayed significant p values (p < 0.05) (Additional file 2). The better fit of values to the partial interference model than the no interference model and the observation that HR values at several markers significantly exceeded 66.67% suggested the presence of crossover interference, which is in agreement with previous conclusions for ‘Fortune’ mandarin chromosome II [24].

**Table 1:** **Localisation of centromere positions using multilocus half-tetrad analysis (HTA)**

**LG**	**N**	**Fit ratio NI/PI**	**Centromere location (cM)**	
			**Cent. Position**	**Conf. Interval** ^**a**^
1	13	1.5	60.66	58.82 - 61.64
2	11	6.9	56.87	55.25 - 58.74
3	11	1.8	90.59	89.08 - 93.92
4	13	5.0	16.14	15.12 - 16.82
5	14	1.7	23.12	22.32 - 25.63
6	12	2.4	6.20	4.79 - 6.80
7	11	8.5	96.43	95.02 - 97.56
8	10	1.4	54.21	50.92 - 56.72
9	9	6.9	52.16	50.29 - 53.77

cM: Centimorgans.

N: Number of used markers.

PI: Partial interference model (m = 4), Cx(Co)^4^.

NI: No Interference model.

a. Confidence interval calculated using drop-off method [44].

#### Centromere mapping using multilocus half-tetrad structure analysis

**Figure 3:**
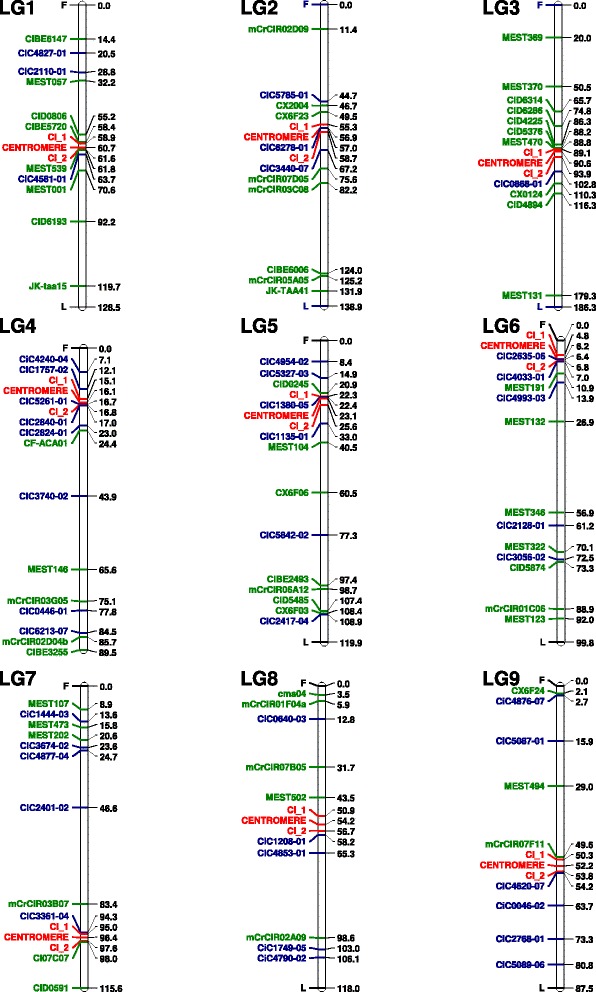
**Location of centromeres on the current clementine genetic map using HTA and drop-off calculations of the confidence interval.** Markers F and L correspond to the first and the last marker for each LG. Green and blue indicate SSR and SNP markers, respectively. Locations of centromeres and CI are highlighted in red.

Having obtained a preliminary location for the centromere in each LG, we selected four flanking markers (two markers on the right and two on the left side of this location) to perform HTA. Four markers were predicted to produce 16 different multilocus profiles, considering homozygosity and heterozygosity at each locus. The number of 2n gametes that corresponded to each of these profiles (Additional file 5) was used to locate the centromere of each LG (Table 1; Figure 3). The 95% CI was calculated using the LOD drop-off method [44].

The centromeres of LGs 1, 3, and 8 were located approximately in the middle of the LG whereas those of LGs 2, 8 and 9 were positioned off-centre, being slightly closer to one end of the LG than the other. In LGs 4, 5, 6 and 7, the centromeres were located very close to one of the LG ends (Figure 3). This genetic mapping of the position of the centromere in each linkage group paves the way for accurate genetic analysis [9,29] and will, in particular, greatly simplify analysis of the mechanisms of 2n gamete formation in different *Citrus* varieties. Very simple, routine tests that identify the mechanism of 2n gamete formation can be performed at the individual level using co-dominant centromeric markers, as previously demonstrated using the *Pgm-2* locus in potatoes [25,65]. In this paper, however, knowledge of the centromere locations was used to analyse the recombination patterns in 2n and haploid clementine gametes, as described below.

### Crossovers and interference analysis in 2n gametes

‘Crossover interference’ refers to the observation that the occurrence of one crossover affects the likelihood of occurrence and/or the location of other crossovers in its neighbourhood [66]. Partial crossover interference in ‘Fortune’ mandarin was proposed by Cuenca *et al.* [24]. In the current study, theoretical partial crossover interference models were a better fit to the patterns of HR observed along all the clementine LGs, as some markers displayed HR values greater than the 66.6% threshold for the no interference model. Using the genetic location of the centromere estimated by HTA, we studied the crossovers occurring in each of the chromosome arms (Table 2). The percentage of 2n gametes that showed multiple crossovers (MCO) in a particular region ranged from 0% (arm 1 of chromosomes IV and V) to 44.16% (arm 2 of chromosome VI). A maximum of four crossovers (CO) was observed; this was seen in arm 1 of chromosome VII and in arm 2 of chromosomes II, IV and VI (Additional file 6). Complementary crossovers (double crossovers implying the presence of four chromatids; CCO) were identified by the occurrence of an allelic phase change in homozygosity between markers (Additional file 7), according to Cuenca *et al.* (24). The maximum percentage of CCO was 19.48%, which was observed both in arm 2 of chromosome VI and in arm 1 of chromosome VII (Table 2).

**Table 2:** **Multiple and complementary crossovers observed in 2n gametes, estimation of the interference coefficient (IC) for each chromosome arm and test for interference between arms (TI)**

**LG**	**Arm 1**			**Arm 2**			**Ratio IC1/IC2**	**TI**	
	**% MCO**	**% CCO**	**IC 1**	**% MCO**	**% CCO**	**IC 2**		**X** ^**2**^	**p-value**
1	6.17	6.17	0.82	22.22	6.17	0.48	1.71	12.36	0.194
2	4.76	0.00	0.60	21.43	15.48	0.53	1.13	5.82	0.667
3	15.48	10.71	0.59	13.10	4.76	0.53	1.11	5.55	0.937
4	0.00	0.00	1.00	22.78	10.13	0.56	1.79	2.73	0.603
5	0.00	0.00	1.00	30.56	19.44	0.38	2.63	1.39	0.708
6	-	-	-	44.16	19.48	0.19		-	-
7	33.77	19.48	0.39	1.30	1.30	1.00	0.39	14.33	0.074
8	8.86	3.80	0.75	16.46	3.80	0.43	1.74	2.49	0.869
9	7.50	2.50	0.62	0.00	0.00	1.00	0.62	2.64	0.268

% MCO: Percentage of 2n gametes with multiple crossovers.

% CCO: Percentage of 2n gametes with complementary crossovers (phase change).

IC: Interference coefficient.

The interference coefficient (IC) was estimated for each chromosome arm. Values of IC ranged considerably from 0.19 (arm 1 of chromosome VI) to 1.0 (arm 1 of chromosomes IV and V and arm 2 of chromosomes VII and IX). Three of the four chromosome arms displaying total interference were very short genetic arms; partial interference was observed in the longer arm of chromosomes V and VII. Different levels of interference between arms of the same LG have been previously reported for chromosome II of ‘Fortune’ mandarin [24]. Potential interference between arms was tested for each chromosome by χ^2^-analysis, based on the contingency table of the number of crossovers in each chromosome arm (Additional file 7), but no interference between the different arms of the same chromosome was observed (Table 2).

Crossover interference plays an important role in determining the number of crossovers per chromosome, but little is known about the mechanisms controlling interference [67]. In clementine, we found no evidence for interference between different arms of a chromosome, a maximum number of four crossovers per chromosome (chromosomes II, IV, VI and VII) and observed total interference in one arm of four different chromosomes (IV, V, VII and IX). Variation in the level of interference between different parts of the genome has been observed in Arabidopsis [68], in humans [69] and in mice [70]. The last work also suggested that levels of interference are also higher in the smaller chromosomes of mice.

### Pattern of recombination in clementine haploid gametes ad its relation with centromere location and genic sequences distribution

**Figure 4:**
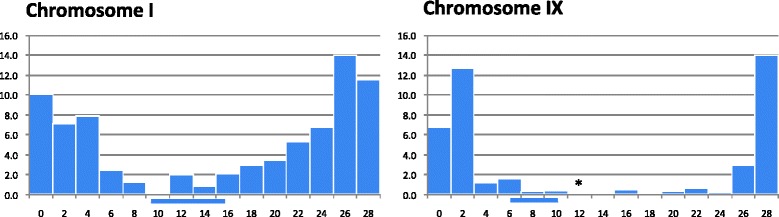
**Variation in recombination rates along chromosomes I and IX of clementine.** The x-axis displays the physical position in megabases (Mb) along each chromosome, and the y-axis represents the ratio of genetic distance to physical distance (cM/Mb). The bars beneath the x-axis indicate the approximate locations of the centromeres (CI). *These data have been calculated from up and down intervals (no marker on the genetic map on the considered genomic segment).

The data from the clementine genetic map [31] were anchored on the whole genome sequence of clementine [32] and used to analyse the pattern of recombination along each chromosome and its relationship with centromere location (Figure 4 and Additional file 8). The cM/Mb rates were estimated using local linear regression between the genetic and physical positions of markers in the region under consideration. The physical locations of the confidence intervals around genetic positions of centromeres were also inferred using local regression between the genetic and physical positions of the flanking markers. From this inference of the physical location of their centromeres, the nine clementine chromosomes should be classified as metacentric (I, II, V and VIII), submetacentric (III, IV, VII and IX) and acrocentric chromosomes (VI), according to the criteria of Levan *et al.* [71].

**Figure 5:**
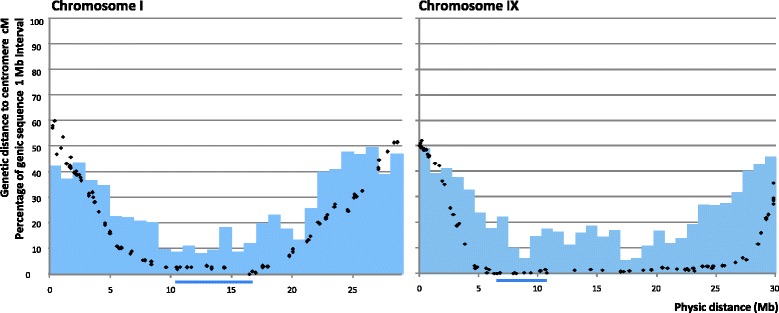
**Relationship between physical location (x-axis), genetic distances between the centromere and markers on the clementine genetic map (black dots) and proportion of genic sequences (blue bars) along chromosomes I and IX.** Bars beneath the x-axis indicate the approximate location of the centromeres (CI).

The average recombination rate over the entire *Citrus* genome was around 3.0 cM/Mb, but there was large variation across the genome. The higher local rates of recombination were less than 14 cM/Mb in most chromosomes, with only chromosomes VI and VIII displaying regions with elevated recombination rates of 26 cM/Mb and 33 cM/Mb, respectively. Centromeric areas displayed very low levels of recombination (<1.0 cM/Mb), but the distance over which this reduction in the rate recombination extended varied considerably between chromosomes. The genomic region with a genetic distance from the centromere of under 5.0 cM was less than 13 Mb for chromosomes I, II, IV, VI and VII, but extended to 23 Mb for chromosome IX and to 30 Mb for chromosome III (Figure 5 and Additional file 9). The fraction of the entire genome within 5.0 cM of a centromere was around 47% but, again, this value varied considerably between chromosomes, being lowest (24%) for chromosome VII and highest (74%) for chromosome IX. Low values of recombination close to the centromeres were first reported by Dobzhansky [72] in *Drosophila melanogaster*. Suppression of crossovers in centromeric and pericentromeric regions has been observed in many plant species, including tomato [73], wheat [74], Arabidopsis [75], rice [76], maize [77] and soybean [71], and this reduction ranges from 5-fold to > 200-fold, depending on the organism [78].

Most chromosomes showed a strong positive correlation between the genetic distance from the centromere and the frequency of genic sequences (Figures 4 and 5 and Additional files 8 and 9). As a consequence, across the whole genome, the 47% of the genome situated within 5 cM of a centromere contained only 23% of the genic sequences. On chromosome IX, however, because the recombination rate was suppressed over a much larger region (Figure 5), 48% of genic sequences were located within 5 cM of the centromere. Chromosome VII displayed a unique pattern, as both the rate of recombination and the frequency of genic sequences were relatively homogeneous across its length. A low frequency of genes in centromeric and pericentromeric regions has been described previously for many plant species [79]. In wheat, for example, most genes are clustered in the distal regions of chromosomes and the large centromeric regions (as large as 100 Mb) are gene-poor and recombinationally inactive [74,80,81]. Outside the centromeric and pericentromeric regions, the relationship between gene density and recombination rate may vary. In rice, Tian *et al.* [82] observed a positive correlation between genomic recombination rates and gene densities, but such a relationship was not found in wheat [81]. In *Citrus*, in addition to containing low densities of genes, the centromeric and pericentromeric areas are characterised by a high frequency of retrotransposon DNA [32]; a similar pattern has been observed in wheat [83]. In the current study, the suppression of crossovers extended beyond the centromeric and pericentromeric regions in chromosome IX and, to a lesser extent, in chromosome VIII.

The interspecific heterozygosity of the clementine genome [32] is likely to be a contributing factor to the level of crossover suppression beyond the centromeric and pericentromeric regions. As a consequence, high levels of linkage disequilibrium will be maintained across large regions of each of these chromosomes, affecting numerous genes. Linkage disequilibrium across extended portions of the genome serves to maintain epistatic interactions and retain phenotypic traits determined by the interactions of specific alleles at multiple loci but, simultaneously, strongly limits the possibility of generating recombined lines and hence new, potentially more favourable, genotypes. Moreover, as 2n gametes of clementine are produced by SDR, many genes on these chromosomes will be transmitted in a homozygous state.

## Conclusions

Triploid hybrid progeny derived from ‘Fina’ clementine × ‘Nadorcott’ tangor crosses were shown to originate from unreduced diploid megagametophytes produced by SDR. Under this model, multilocus HTA was used to map genetically the position of the centromere in all nine *Citrus* linkage groups. Identification of the centromere positions paves the way for developing simple methods to determine the mechanism of unreduced gamete formation in a large range of genotypes. Understanding how the distance between genes and centromeres varies across an entire genome, and the effect of sexual polyploidisation, will enable better modelling of the inheritance of favourable traits and thus enhance breeding schemes.

The inference of the physical location of the centromeres from the whole genome sequence of *Citrus* anchored on the genetic map revealed one acrocentric, four metacentric and four submetacentric chromosomes. Partial crossover interference was observed in at least one arm of each chromosome, with variation between arms. Total interference was found in four chromosome arms, although no interference was observed between the different arms of the same chromosome. For haploid clementine gametes, a decrease in the rate of recombination was observed in centromeric and pericentromeric regions, which contained a low density of genic sequences. On chromosomes VIII and IX, these regions of limited recombination extended beyond the pericentromeric regions and affected areas richer in genic sequences. This should produce strong linkage disequilibrium for a large number of genes, resulting in stable epistatic interactions and retention of multilocus-controlled traits over successive generations, but will also maintain multi-trait associations. The genomic region corresponding to a genetic distance less than 5 cM from a centromere ranged from 5.0 Mb in chromosome VII to 30 Mb in chromosome III. In total, this represented 47% of the *Citrus* genome and 23% of the genic sequences. These findings will be helpful for optimising breeding strategies based on sexual hybridisation and also for better understanding the genetics of triploid *Citrus*.

## Additional files

Additional file 1:
**Characteristics and genetic/physical positions of the SSR, InDel and SNP markers used in mapping of triploid progenies to determine the positions of the centromeres.**
Additional file 2:
**χ** ^**2**^ **-test for allelic segregation and heterozygosity restitution percentages (%HR) at each locus, LOD SDR/FDR for markers with HR < 50% and χ** ^**2**^ **-test for partial interference for markers with HR > 66.67%.**
Additional file 3:
**Analysis at individual level of the origin (SDR/FDR) of clementine 2n gametes.** For each of the nine LGs, we selected the marker closest to the estimated position of the centromere and computed the LOD of the probability of the individual multilocus allelic configuration under SDR/FDR hypotheses (Cuenca *et al*., [24]). No more than one marker in heterozygosity was observed for each individual and the LOD value supported SDR as the origin of the 2n gamete. Additional file 4:
**Observed heterozygosity restitution (HR frequency) values for markers (squares) and theoretical functions for SDR with partial interference (x-axis indicates Kosambi’s distance) and no interference models (x-axis indicates Haldane’s distance).**
Additional file 5:
**Heterozygous and homozygous profiles for 2n gametes analysed by HTA using four markers flanking the position of the centromere in each LG.** N: Number of triploid hybrids for each profile; M: Name of the molecular marker; HR%: Heterozygosity restitution value; HE: Heterozygous; HO: Homozygous. Additional file 6:
**Distribution of the number of crossovers observed in 2n gametes on each arm of each LGs.**
Additional file 7:
**Analysis of crossover interference in clementine. Heterozygous (HE, ab) and homozygous (HO, aa and bb) profiles of 2n gametes analysed using 11 markers.** The location of the centromere on LG 3 was estimated using HTA. Numbers in grey are the number of 2n gametes with different crossover events on one arm and between arms (0, 1, 2, 3 and 4). Numbers in bold correspond to percentages of crossovers observed in each chromosome arm. Potential interference between arms was tested for each chromosome using χ^2^-analysis based on the contingency table of the number of crossovers (grey numbers) in each chromosome arm. Additional file 8:
**Variation in recombination rate along the different clementine chromosomes (Chr).** The x-axis shows the physical position in megabases along each chromosome and the y-axis represents the ratio of genetic distance to physical distance (cM/Mb). The bars beneath the x-axis indicate the approximate locations of the centromeres (CI). * These data have been calculated from up and down intervals (no marker on the genetic map on the considered genomic segment). Additional file 9:
**Relationship between the physical location (x-axis), the genetic distance between centromere and markers on the clementine genetic map (black dots) and the proportion of gene sequences (blue bars) along all chromosomes.** Bars beneath the x-axis indicate the approximate locations of the centromeres location (CI).

## Abbreviations

Chr: Chromosome
CCO: Complementary crossovers
FDR: First-division restitution
HTA: Half-tetrad analysis
HR: Heterozygosity restitution
InDel: Insertion-deletion
IC: Interference coefficient
LG: Linkage group
LOD: Logarithm of the odds ratio
MCO: Multiple crossovers
SDR: Second-division restitution
SSR: Simple sequence repeat
SNP: Single nucleotide polymorphism

## References

[1] Hall AE, Keith KC, Hall SE, Copenhaver GP, Preuss D (2004). The rapidly evolving field of plant centromeres. Curr Opin Plant Biol.

[2] Copenhaver GP, Nickel K, Kuromori T, Benito MI, Kaul S, Lin X (1999). Genetic definition and sequence analysis of Arabidopsis centromeres. Science.

[3] Ma J, Wing RA, Bennetzen JL, Jackson SA (2007). Plant Centromere organization: a dynamic structure with conserved functions. Trends Genet.

[4] Jiang J, Birchler JA, Parrot WA, Dawe RK (2003). A molecular view of plant centromeres. Trends Plant Sci.

[5] Houben A, Schubert I (2003). DNA and proteins of plant centromeres. Curr Opin Plant Biol.

[6] Nie H, Li Q, Zhao X, Kong L (2013). Genetic positioning of centromeres through half-tetrad analysis in gynogenetic diploid families of the Zhikong Scallop (*Chlamysfarreri*). Mar Biotechnol.

[7] Singh K, Ishii T, Parco A, Huang N, Brar DS, Khush GS (1996). Centromere mapping and orientation of the molecular linkage map of rice (Oryzasativa L). PNAS.

[8] Guyomard R, Mauger S, Tabet-Canale K, Martineau S, Genet C, Krieg F (2006). A type I and type II microsatellite linkage map of rainbow trout (Oncorhynchusmykiss) with presumptive coverage of all chromosome arms. BMC Genomics.

[9] Park TH, Kim JB, Hutten RCB, van Eck HJ, Jacobsen E, Visser RGE (2007). Genetic positioning of centromeres using half-tetrad analysis in a 4x-2x cross population of potato. Genetics.

[10] Copenhaver GP, Keith KC, Preuss D (2000). Tetrad analysis in higher plants. a budding technology. Plant Physiol.

[11] Harlan JR, De Wet JMJ (1975). On O. Winge and a prayer: the origins of polyploidy. Botan Rev.

[12] Bretagnolle F, Thompson JD (1995). Gametes with the somatic chromosome number: mechanisms of their formation and role in the evolution of autopolyploid plants. New Phytol.

[13] Ramsey J, Schemske DW (1998). Pathways, mechanisms, and rates of polyploidy formation in flowering plants. Annu Rev Ecol Syst.

[14] Ramsey J, Schemske DW (2002). Neopolyploidy in flowering plants. Annu Rev Ecol Syst.

[15] Tavoletti S, Bingham ET, Yandell BS, Veronesi F, Osborn TC (1996). Half tetrad analysis in alfalfa using multiple restriction fragment length polymorphism markers. Proc Natl Acad Sci U S A.

[16] Rhoades MM, Dempsey E (1966). Induction of chromosome doubling at meiosis by the elongate gene in maize. Genetics.

[17] Mendiburu AO, Peloquin SJ (1979). Gene-centromere mapping by 4x-2x matings in potatoes. Theor Appl Genet.

[18] Veilleux R (1985). Diploid and polyploid gametes in crop plants: mechanisms of formation and utilization in plant breeding. Plant Breed Rev.

[19] Carputo D, Barone A, Frusciante L (2000). 2n gemetes in the potato: essential ingredients for breeding and germplasm transfer. Theor Appl Genet.

[20] Ramanna MS, Jacobsen E (2003). Relevance of sexual polyploidization for crop improvement—a review. Euphytica.

[21] Esen A, Soost RK (1971). Unexpected triploids in citrus: their origin, identification and possible use. J Hered.

[22] Geraci G, De Pasquale F, Tusa N, Grierson W (1977). Percentages of spontaneous triploids in progenies of diploid lemons and mandarins. Proceedings of Second International Citrus Congress.International Society of Citriculture.

[23] Bastiaanssen HJM, Van Den Berg PMMM, Lindhout P, Jacobsen E, Ramanna MS (1998). Postmeiotic restitution in 2n-egg formation of diploid potato. Heredity.

[24] Cuenca J, Froelicher Y, Aleza P, Juarez J, Navarro L, Ollitrault P (2011). Multi*locus* half-tetrad analysis and centromere mapping in citrus: evidence of SDR mechanism for 2n megagametophyte production and partial chiasma interference in mandarin cv ‘Fortune’. Heredity.

[25] Douches DS, Quiros CF (1988). Genetic strategies to determine the mode of 2n egg formation in diploid potatoes. Euphytica.

[26] Luro F, Maddy F, Ollitrault P, Rist D (2000). Identification of 2n gamete parental origin and mode of nuclear restitution of spontaneous triploid citrus hybrids. Proceedings of 9th International Citrus Congress.

[27] Crespel L, Gudin S (2003). Evidence for the production of unreduced gametes by tetraploid Rosa hybrida L. Euphytica.

[28] Kauffman EJ, Gestl EE, Kim DJ, Walker C, Hite JM, Yan G (1995). Microsatellite–centromere mapping in the Zebrafish (Daniorerio). Genomics.

[29] Bastiaanssen HJM, Ramanna MS, Sawor Z, Mincione A, Steen A, Jacobsen E (1996). Pollen markers for gene–centromere mapping in diploid potato. Theor Appl Genet.

[30] FAOSTAT [http://faostat.fao.org/site/567/DesktopDefault.aspx?PageID=567#ancor]

[31] Ollitrault P, Terol J, Chen C, Federici CT, Lotfy S, Hippolyte I (2012). A reference genetic map of C. clementina hort. ex Tan.; citrus evolution inferences from comparative mapping. BMC Genomics.

[32] Wu AG, Prochnik S, Jenkins J, Salse J, Hellsten U, Murat F (2014). Sequencing of diverse mandarin, pummelo and orange genomes reveals complex history of admixture during citrus domestication. Nat Biotechnol.

[33] Aleza P, Juárez J, Hernández M, Pina JA, Ollitrault P, Navarro L (2009). Recovery and characterization of a Citrus clementina Hort ex Tan Clemenules haploid plant selected to establish the reference whole Citrus genome sequence. BMC Plant Biol.

[34] Ollitrault P, Dambier D, Luro F, Froelicher Y (2008). Ploidy manipulation for breeding seedless triploid citrus. Plant Breed Rev.

[35] Aleza P, Juárez J, Cuenca J, Ollitrault P, Navarro L (2010). Recovery of citrus triploid hybrids by embryo rescue and flow cytometry from 2x X 2x sexual hybridisation and its application to extensive breeding programs. Plant Cell Rep.

[36] Chen C, Lyon MT, O’Malley D, Federici CT, Gmitter J, Grosser JW (2008). Origin and frequency of 2n gametes in Citrus sinensis x Poncirus trifoliata and their reciprocal crosses. Plant Sci.

[37] Ollitrault P, Terol J, Garcia-Lor A, Bérard A, Chauveau A, Froelicher Y (2012). SNP mining in C. clementina BAC end sequences; transferability in the Citrus genus (Rutaceae), phylogenetic inferences and perspectives for genetic mapping. BMC Genomics.

[38] Esselink GD, Nybom H, Vosman B (2004). Assignment of allelic configuration in polyploids using the MAC-PR (microsatellite DNA allele counting-peak ratios) method. Theor Appl Genet.

[39] Cuppen E. Genotyping by allele-specific amplification (KASPar). Cold Spring Harb Protoc 2007; 172–173. doi: 10.1101/pdb.prot4841. 10.1101/pdb.prot4841 21357174

[40] Cuenca J, Aleza P, Vicent A, Brunel D, Ollitrault P, Navarro L (2013). Genetically based location from triploid populations and gene ontology of a 3.3-Mb genome region linked to alternaria brown spot resistance in citrus reveal clusters of resistance genes. PLoS One.

[41] Zhao H, Speed TP (1998). Statistical analysis of ordered tetrads. Genetics.

[42] Griffiths A, Miller J, Suzuki D, Lewontin R, Gelbart W (1996). An Introduction to Genetic Analysis.

[43] Zhao H, Speed TP (1998). Statistical analysis of half-tetrads. Genetics.

[44] Lander ES, Botstein D (1989). Mapping Mendelian factors underlying quantitative traits using RFLP linkage maps. Genetics.

[45] Altschul SF, Gish W, Miller W, Myers EW, Lipman DJ (1990). Basic local alignment search tool. J Mol Biol.

[46] Esen A, Soost RK (1973). Precocious development and germination of spontaneous triploid seeds in citrus. J Hered.

[47] Geraci G, Esen A, Soost RK (1975). Triploid progenies from 2x–2x crosses of citrus cultivars. J Hered.

[48] Esen A, Soost RK, Geraci G (1979). Genetic evidence for the origin of diploid megagametophytes in Citrus. J Hered.

[49] Wakana A, Iwamasa M, Uemoto S (1981). Seed development in relation to ploidy of zygotic embryo and endosperm in polyembryonic Citrus. Proc Int Soc Citricult.

[50] Soost R, Abbott AJ, Atkin R (1987). Breeding citrus-genetics and nucellarembryony. Improving Vegetatively Propagated Crops.

[51] Ferrante SP, Lucretti S, Reale S, De Patrizio A, Abbate L, Tusa N (2010). Assessment of the origin of new citrus tetraploid hybrids (2n = 4x) by means of SSR markers and PCR based dosage effects. Euphytica.

[52] Errico A, Cammareri M, Conicella C (2005). Meiotic nuclear restitution mechanisms in a triploid lily. Caryologia.

[53] Brownfield L, Kohler C (2011). Unreduced gamete formation in plants: mechanisms and prospects. J Exp Bot.

[54] Barone A, Gebhardt C, Frusciante L (1995). Heterozygosity in 2n gametes of potato evaluated by RFLP markers. Theor Appl Genet.

[55] Vorsa N, Rowland LJ (1997). Estimation of 2n megagametophyteheterozygosity in a diploid blueberry (Vacciniumdarrowi Camp) clone using RAPDs. J Hered.

[56] Dewitte A, Van Laere K, Van Huylenbroeck J, Ibrokhim Y (2011). Use of 2n fametes in plant breeding. Plant Breeding.

[57] Douches DS, Maas DL (1998). Comparison of FDR and SDR derived tetraploid progeny from 2x x 4x crosses using haploids of Solanumtuberosum L. that produce mixed modes of 2n eggs. Theor Appl Genet.

[58] Mok DWS, Peloquin SJ (1975). Three mechanisms of 2n pollen formation in diploid potatoes. Can J Genet Cytol.

[59] Kidane-Mariam HM, Arndt GC, Macaso-Khwaja AC, Peloquin SJ (1985). Comparisons between 4x × 2x hybrid and open-pollinated true potato seed families. Potato Res.

[60] David JL, Boudec P, Gallais A (1995). Quantitative genetics of 4x-2x hybrid populations with First-Division Restitution and 2nd-Division Restitution 2n gametes produced by diploid parents. Genetics.

[61] Buso JA, Boiteux LS, Tai GCC, Peloquin SJ (1999). Chromosome regions between centromeres and proximal crossovers are the physical sites of major effect loci for yield in potato: genetic analysis employing meiotic mutants. PNAS.

[62] Zhu C, Sun Y, Yu X, Tong J (2013). Centromere localization for bighead carp (Aristichthysnobilis) through Half-Tetrad analysis in diploid gynogenetic families. PLoS One.

[63] Zhao H, Speed TP, McPeek MS (1995). Statistical analysis of crossover interference using the chi-square model. Genetics.

[64] Zhao H, Speed TP (1996). On genetic map functions. Genetics.

[65] Werner JE, Douches DS, Freyre R (1992). Use of half-tetrad analysis to discriminate between 2 types of 2n egg formation in a potato haploid. Genome.

[66] Teuscher F, Brockmann GA, Rudolph PE, Swalve HH, Guiard V (2000). Models for chromatid interference with applications to recombination data. Genetics.

[67] Giraut L, Falque M, Drouaud J, Pereira L, Martin OC, Mézard C. Genome-Wide crossover distribution in arabidopsis thaliana meiosis reveals sex-specific patterns along chromosomes. Plos Genetics 2011, doi:10.1371/journal.pgen.1002354. 10.1371/journal.pgen.1002354 PMC3207851 22072983

[68] Drouaud J, Mercier R, Chelysheva L, Berard A, Falque M, Martin O (2007). Sex-specific crossover distributions and variations in interference level along Arabidopsis thaliana chromosome 4. PLoS Genet.

[69] Lian J, Yin Y, Oliver-Bonet M, Liehr T, Ko E, Turek P (2008). Variation in crossover interference levels on individual chromosomes from human males. Hum Mol Genet.

[70] Broman KW, Rowe LB, Churchill GA, Paigen K (2002). Crossover interference in the mouse. Genetics.

[71] Levan A, Fredga K, Sandberg AA (1964). Nomenclature for centromeric position on chromosomes. Hereditas.

[72] Dobzhansky T (1930). Translocations involving the third and fourth chromosomes of Drosophila melanogaster. Genetics.

[73] Sherman JD, Stack SM (1995). Two-dimensional spreads of synaptonemal complexes from solanaceous plants VI high-resolution recombination nodule map for tomato (Lycopersiconesculentum). Genetics.

[74] Gill SK, Gill BS, Endo TR, Taylor T (1996). Identification and high-density mapping of gene-rich regions in chromosome group I of wheat. Genetics.

[75] Haupt W, Fischer TC, Winderl S, Fransz P, Torres-Ruiz RA (2001). The centromere1 (CEN1) region of Arabidopsis thaliana: architecture and functional impact of chromatin. Plant J.

[76] Chen M, Prestin G, Barbazuk WB, Goicoechea JL, Blackmon B, Fang G (2002). An integrated physical and genetic map of the rice genome. Plant Cell.

[77] Anderson LK, Doyle GG, Brigham B, Carter J, Hooker KD, Lai A (2003). High resolution crossover maps for each bivalent of Zea mays using recombination nodules. Genetics.

[78] Talbert PB, Henikoff S (2010). Centromeres convert but don’t cross. PLoS Biol.

[79] Wang G, Zhang X, Jin W (2009). An overview of plant centromeres. J Genet Genomics.

[80] Sandhu D, Gill K (2002). Gene-containing regions of wheat and the other grass genomes. Plant Physiol.

[81] Erayman M, Sandhu D, Sidhu D, Dilbirligi M, Baenziger PS, Gill KS (2004). Demarcating the gene-rich regions of the wheat genome. Nucleic Acids Res.

[82] Tian Z, Rizzon C, Du J, Zhu L, Bennetzen JL, Jackson SA (2009). Do genetic recombination and gene density shape the pattern of DNA elimination in rice long terminal repeat retrotransposons?. Genome Res.

[83] Liu Z, Yue W, Li D, Wang R, Kong X, Lu K (2008). Structure and dynamics of retrotransposons at wheat centromeres and pericentromeres. Chromosoma.

