# Supplementary material for: Genetic mapping of centromeres in the nine Citrus clementina chromosomes using half-tetrad analysis and recombination patterns in unreduced and haploid gametes
Source: BMC Plant Biol. 2015 Mar 8;15:80. doi: 10.1186/s12870-015-0464-y (PMC4367916; doi:10.1186/s12870-015-0464-y)
Supplement: Additional file 1: — Characteristics and genetic/physical positions of the SSR, InDel and SNP markers used in mapping of triploid progenies to determine the positions of the centromeres. [file 12870_2015_464_MOESM1_ESM.pdf]

Additional file 1. Characteristics and genetic/physical positions of the SSR, InDel and SNP markers used in mapping of triploid progenies to determine the positions of the centromeres

Marker	Gene Bank Accesion	Linkage Group	Genetic position (cM);[31]	Physic position (nt); [32]	Alleles [1]		Bibliographic reference
					Fina	Nadorcott	
CIBE6147	ET085226	1	14,4	1755414	205 - 213	207 - 213	[93]
CiC4827-01	ET072918	1	20,54	2036846	GA	AA	[37]
CiC2110-01	ET099643	1	28,82	3241022	CA	AA	[37]
MEST057	DY267931	1	32,16	3730905	218 - 221	218 - 218	In preparation
CID0806	ET094469	1	55,17	7860474	368 - 380	371 - 380	[94]
CiBE5720	ET082224	1	58,45	13332543	325 - 336	329 - 336	[93]
MEST539	DY294904	1	61,82	16725002	104 - 108	104 - 104	In preparation
CiC4581-01	ET109034	1	63,66	17994845	AC	AA	[37]
MEST001	DY262452	1	70,61	20040617	171 - 175	171 - 171	[92]
CiC5950-02	ET083949	1	91,37	25122291	GA	GG	[37]
CID6193	ET085125	1	92,22	25132488	176 - 185	176 - 176	[94]
CiC0599-01	ET093125	1	102,39	27107805	GA	GG	[37]
JK-taa15	none	1	119,73	28183310	189 - 192	189 - 204	[91]
mCrCIR02D09	FR677569	2	11,37	5821301	231 - 238	229 - 231	[24]
CiC5785-01	ET082673	2	44,73	11235057	TA	TT	[37]
Cx2004	CF653229	2	46,67	11397075	200 - 204	204 - 204	[87]
Cx6F23	CF417259	2	49,53	11488134	149 - 162	162 - 168	[86]
CiC6278-01	ET085551	2	57,01	20807016	CA	AA	[37]
CiC3440-07	ET077539	2	67,22	24636345	GA	GG	[37]
mCrCIR07D05	FR677574	2	75,6	26516983	188 - 190	186 - 188	[24]
mCrCIR03C08	FR677576	2	82,19	27340389	207 - 226	207 - 208	[24]
CIBE6006	ET084205	2	124,01	34151440	176 - 201	197 - 201	[93]
mCrCIR05A05	FR677580	2	125,24	34232586	144 - 153	153 - 164	[24]
JK-taa41	none	2	131,86	35861149	146 - 154	154 - 160	[91]
MEST369	DY272147	3	20	3587345	153 - 157	153 - 160	In preparation
MEST370	DY295074	3	50,54	6159065	181 - 184	181 - 181	In preparation
CID6314	ET085782	3	65,71	7424910	231 - 240	240 - 240	[94]
CID6286	ET085618	3	74,76	9132118	365 - 384	365 - 365	[94]
CIBE4225	ET081679	3	86,33	32288952	132 - 144	132 - 132	This paper 2
CID5376	ET113309	3	88,24	12826450	169 - 174	174 - 174	[94]
MEST470	DY290454	3	88,76	11399251	255 - 257	255 - 255	In preparation
CiC0868-01	ET095107	3	102,81	38496917	CT	CC	[37]
CX0124	CN187496	3	110,28	41286578	181 - 190	181 - 181	Chen et al In preparation
CID4894	ET080676	3	116,28	42870140	220 - 227	220 - 220	[94]
MEST131	DY276912	3	179,33	50550369	141 - 147	141 - 141	[89]
CiC4240-04	ET106812	4	7,09	2315148	GA	GG	[37]
CiC1757-02	ET097717	4	12,05	3714825	CA	CC	[37]
CiC5261-01	ET112543	4	16,65	7935718	TA	TT	[37]
CiC2840-01	ET103429	4	16,96	9827893	CT	TT	[37]
CiC2824-01	ET103336	4	22,97	14673848	AG	AA	[37]
CF-ACA01	CN181701.1	4	24,41	14526637	336 - 339	336 - 336	In preparation
CiC3740-02	ET079647	4	43,95	18980142	GC	CC	[37]
MEST146	DY278930	4	65,61	22394239	231 - 237	231 - 231	In preparation
mCrCIR03G05	FR677578	4	75,06	23443938	226 - 229	226 - 226	[24]
CiC0446-01	ET091387	4	77,76	23905799	TA	TT	[37]
CiC6213-07	ET085253	4	84,53	24871638	GA	GG	[37]
mCrCIR02D04b	FR677564	4	85,74	25569961	200 - 210	200 - 230	[90]
CIBE3255	ET076159	4	89,49	24918812	205 - 210	210 - 210	[93]
CiC4954-02	ET081013	5	8,41	1604845	GA	GG	[37]
CiC5327-03	ET113032	5	14,9	6430315	CT	CC	[37]
CID0245	ET089411	5	20,94	22294452	209 - 216	216 - 216	This paper 3
CiC1380-05	ET072553	5	22,42	10987419	TC	CC	[37]
CiC1135-01	ET069608	5	32,96	29766765	GA	AA	[37]
MEST104	DY273697	5	40,46	32963594	238 - 242	236 - 238	[89]
CiC5788-16	ET082679	5	41,54	33591652	GA	AA	[37]

Cx6F06	CK933007	5	60,5	35908207	150 - 153	150 - 150	[86]
CiC5842-02	ET083106	5	77,34	37109366	CA	CC	[37]
CID2493	ET101792	5	96,68	38674890	173 - 178	169 - 171	[94]
mCrCIR06A12	AM489742	5	98,71	39956736	97 - 102	97 - 97	[88]
CID5485	ET113963	5	107,43	41715026	229 - 235	229 - 229	[94]
Cx6F03	DR912164	5	108,4	42010039	254 - 260	248 - 248	[86]
CiC2417-04	ET101382	5	108,94	41973469	TA	TT	[37]
CiC2635-06	ET102138	6	6,38	9585251	AG	GG	[37]
CiC4033-01	ET105470	6	7,04	8322694	TA	TT	[37]
MEST191	DY283044	6	10,86	11114011	241 - 243	235 - 243	In preparation
CiC4993-03	ET081280	6	13,95	12258804	CT	CC	[37]
MEST132	DY276930	6	26,93	15158795	235 - 262	235 - 244	[85]
MEST346	DY291500	6	56,94	19933128	277 - 297	297 - 297	In preparation
CiC2128-01	ET111354	6	61,19	19377331	CA	CC	[37]
MEST322	DY297861	6	70,14	21781641	164 - 180	180 - 180	In preparation
CiC3056-02	ET075329	6	72,51	22086724	GA	AA	[37]
CID5874	ET083265	6	73,33	22257939	376 - 382	382 - 392	[94]
mCrCIR01C06	FR692356	6	88,92	24791517	133 - 165	131 - 159	[24]
MEST123	DY276100	6	91,98	25303966	253 - 281	253 - 270	[85]
MEST107	DY274062	7	8,9	211621	176 - 184	176 - 176	[89]
CiC1444-03	ET073216	7	13,6	562320	CA	CC	[37]
MEST473	DY295492	7	15,77	784405	218 - 226	Null - 228	In preparation
MEST202	DY284147	7	20,6	1941762	157 - 169	Null - 169	In preparation
CiC3674-02	ET079224	7	23,56	2639362	GA	GG	[37]
CiC4877-04	ET080581	7	24,68	2733388	GA	AA	[37]
CiC2401-02	ET101266	7	46,63	7084364	AG	AA	[37]
mCrCIR03B07	FR677573	7	83,39	11545990	264 - 266	266 - Null	[24]
CiC3361-04	ET076974	7	94,28	15356223	GT	GNull	[37]
Ci07C07	AJ567409	7	98,02	15803823	228 - 240	225 - 240	[88]
CID0591	ET093022	7	115,59	21089419	346 - 349	Null - 355	[94]
CMS04	none	8	3,54	21700	173 - 189	Null - 189	[84]
mCrCIR01F04a	AM489736	8	5,92	1063542	187 - 203	208 - 222	[88]
CiC0640-03	ET068363	8	12,79	1871233	AG	GG	[37]
mCrCIR07B05	AM489747	8	31,7	3217225	203 - 222	217 - 220	[88]
MEST502	DY266990	8	43,45	5153447	175 - 192	175 - 183	In preparation
CiC1208-01	ET070547	8	58,18	18991766	GA	GNull	[37]
CiC4853-01	ET110900	8	65,28	22027045	TC	CN	[37]
mCrCIR02A09	FR677568	8	98,63	24367691	159 - 161	151 - 161	[24]
CiC1749-05	ET097636	8	102,98	24429013	TG	G0	[37]
CiC4790-02	ET110402	8	106,12	24513683	CT	TT	[37]
Cx6F24	CF418081	9	2,11	74436	138 - 144	138 - 138	[86]
CiC4876-07	ET080580	9	2,69	143924	TA	AA	[37]
CiC5087-01	ET111514	9	15,88	1955754	AT	AA	[37]
MEST494	FC882340	9	28,97	2906699	280 - 285	275 - 280	In preparation
mCrCIR07F11	FR677567	9	49,57	4918314	153 - 160	160 - 164	[90]
CiC4620-07	ET109292	9	54,17	23121885	AG	AN	[37]
CiC0046-02	ET087154	9	63,72	28604292	CG	GG	[37]
CiC2768-01	ET102921	9	73,33	29237063	GT	GG	[37]
CiC5089-06	ET111533	9	80,77	29866487	CT	CC	[37]

1: Alleles. The numbers indicate the size of alleles in nucleotides for SSR markers, and letters correspond to SNP markers alleles. Null indicates null alleles.

2: Sequence of primers for SSR marker CIBE4225. Forward: GTGGAGCGATTTGACATT, and Reverse: CTCACAGCAACAACAACAAC.

3: Sequence of primers for SSR marker CID0245. Forward: TGTCTGTTGTTTTGGTGGGCA, and Reverse: GGAAGCAATTATTCCAAGGCCA.

References

24. Cuenca J, Froelicher Y, Aleza P, Juarez J, Navarro L, Ollitrault P: **Multilocus half-tetrad analysis and centromere mapping in citrus: evidence of SDR mechanism for 2n megagametophyte production and partial chiasma interference in mandarin cv 'Fortune'**. *Heredity* 2011, 107: 462-470.

31. Ollitrault P, Terol J, Chen C, Federici CT, Lotfy S, Hippolyte I, Ollitrault F, Bérard A, Chauveau A, Cuenca J, Costantino G, Kacar Y, Mu L, Garcia-Lor A, Froelicher Y, Aleza P, Boland A, Billot C, Navarro L, Luro F, Roose ML, Gmitter FG, Talon M, Brunel D: **A reference genetic map of C. clementina hort. ex Tan.; citrus evolution inferences from comparative mapping**. *BMC Genomics* 13: 593.

32. Wu AG, Prochnik S, Jenkins J, salse J, Hellsten U, Murat F, Perrier X, Ruiz M, Scalabrin S, Terol J, Takita MA, Labadie K, Poulain J, Couloux A, Jabbari K, Cattonaro F, Del Fabbro C, Pinosio S, Zuccolo A, Chapman J, Grimwood J, Tadeo FR, Estornell LH, Muñoz-Sanz JV, Ibañez V, Herrero-Ortega A, Aleza P, Pérez´Pérez J, Ramón D, Brunel D, Luro F, Chen C, Farmerie WG, Desany B, Kodira C, Mohiuddin M, Harkins T, Fredrikson K, Burns P, Lomsadze, Borodovsky M, Reforgiato G, Freitas-Astúa J, Quetier F, Navarro L, Roose M, Wincker P, Schmutz J, Morgante M, Machado MA, Talon m, Jaillon O, Ollitrault P, Gmitter F, Rokhsar D: **Sequencing of diverse mandarin, pummelo and orange genomes reveals complex history of admixture during citrus domestication**. *Nat Biotechnol* 2014, 32:656–662.

37. Ollitrault P, Terol J, Garcia-Lor A, Bérard A, Chauveau A, Froelicher Y, Belzile C, Morillon R, Navarro L, Brunel D, Talon M: **SNP mining in C. clementina BAC end sequences; transferability in the Citrus genus (Rutaceae), phylogenetic inferences and perspectives for genetic mapping**. *BMC Genomics* 2012, 10;13:13

84. Ahmad R, Struss D, Southwick SM: **Development and characterization of microsatellite markers in Citrus.** *J Am Soc Hortic Sci* 2003, 128:584–590.

85. Aleza P, Froelicher Y, Schwarz S, Agusti M, Hernandez M, Juarez J, Luro F, Morillon R, Navarro L, Ollitrault P: **Tetraploidization events by chromosome doubling of nucellar cells are frequent in apomictic citrus and are dependent on genotype and environment**. *Ann Bot* 2011, 108(1):37-50.

86. Chen CX, Zhou P, Choi YA, Huang S, Gmitter FG Jr: **Mining and characterizing microsatellites from citrus ESTs.** *Theor Appl Genet* 2006, 112(7):1248-1257

87. Chen CX, Bowman KD, Choi YA, Dang PM, Rao MN, Huang S, Soneji JR, McCollum TG, Gmitter FG Jr: **EST-SSR genetic maps for Citrus sinensis and Poncirus trifoliata**. *Tree Genet Genomes* 2008, 4(1):1–10.

88. Froelicher Y, Dambier D, Bassene JB, Costantino G, Lotfy S, Didout C, Beaumont V, Brottier P, Risterucci AM, Luro F, Ollitrault P: **Characterization of microsatellite markers in mandarin orange (Citrus reticulata Blanco**). *Mol Ecol Resour* 2008, 8(1):119-122

89. Garcia-Lor A, Luro F, Navarro L, Ollitrault P: **Comparative use of InDel and SSR markers in deciphering the interspecific structure of cultivated citrus genetic diversity: a perspective for genetic association studies**. *Mol Genet Genomics* 2012, 287(1):77–94

90. Kamiri M, Stift M, Srairi I, Costantino G, El Moussadik A, Hmyene A, Bakry F, Ollitrault P, Froelicher Y: **Evidence for non-disomic inheritance in a Citrus interspecific tetraploid somatic hybrid between C. reticulata and C. limon using SSR markers and cytogenetic analysis**. *Plant Cell Rep* 2011, 30(8):1415-1425.

91. Kijas JMH, Thomas MR, Fowler JCS, Roose ML: **Integration of trinucleotide microsatellites into a linkage map of Citrus.** *Theor Appl Genet* 1997, 94(5):701-706.

92. Luro FL, Costantino G, Terol J, Argout X, Allario T, Wincker P, Talon M, Ollitrault P, Morillon R: **Transferability of the EST-SSRs developed on Nules clementine (Citrus clementina Hort ex Tan) to other Citrus species and their effectiveness for genetic mapping.** *BMC Genomics* 2008, 9:287.

93. Ollitrault F, Terol J, Pina JA, Navarro L, Talon M, Ollitrault P: **Development of SSR markers from Citrus clementina (Rutaceae) BAC end sequences and interspecific transferability in Citrus**. *Am J Bot* 2010, 97(11):e124–e129

94. Ollitrault F, Terol J, Alonso Martin A, Pina JA, Navarro L, Talon M, Ollitrault P: **Development of InDel markers from Citrus clementina (Rutaceae) BACend sequences and interspecific transferability in Citrus**. *Am J Bot* 2012, 99(7):e268-e273.
