# Supplementary material for: Genetic mapping of centromeres in the nine Citrus clementina chromosomes using half-tetrad analysis and recombination patterns in unreduced and haploid gametes
Source: BMC Plant Biol. 2015 Mar 8;15:80. doi: 10.1186/s12870-015-0464-y (PMC4367916; doi:10.1186/s12870-015-0464-y)
Supplement: Additional file 2: — χ 2 -test for allelic segregation and heterozygosity restitution percentages (%HR) at each locus, LOD SDR/FDR for markers with HR < 50% and χ 2 -test for partial interference for markers with HR > 66.67%. [file 12870_2015_464_MOESM2_ESM.pdf]

Additional file 2. χ2-test for allelic segregation and heterozygosity restitution percentages (%HR) at each locus, LOD SDR/FDR for markers with HR < 50% and χ2-test for partial interference for markers with HR > 66.67% .

		Allelic segregation			LOD	Partial interference	
**Marker**	**LG**	χ^2	**p (value)**	**% HR**	**SDR/FDR**	χ^2	**p (value)**
CIBE5720	1	0,95	0,33	2,30	22,05		
CIBE6147	1	0,03	0,85	66,67			
CiC0599-01	1	0,03	0,86	63,10			
CiC2110-01	1	0,56	0,46	47,67	0,04		
CiC4581-01	1	0,45	0,50	6,98	16,63		
CiC4827-01	1	0,12	0,73	60,47			
CiC5950-02	1	1,45	0,23	49,43	0,00		
CID0806	1	0,76	0,38	3,45	20,52		
CID6193	1	1,14	0,29	50,57			
JK-taa15	1	0,04	0,84	71,26		0,95	0,33
MEST001	1	0,06	0,81	19,54	7,53		
MEST057	1	1,19	0,28	37,93	1,11		
MEST539	1	0,74	0,39	1,15	23,82		
CIBE6006	2	2,33	0,13	75,86		3,80	0,05
CiC3440-07	2	0,05	0,82	11,63	12,61		
CiC5785-01	2	0,13	0,71	20,24	7,16		
CiC6278-01	2	0,42	0,52	0,00	1,00		
Cx2004	2	0,05	0,82	14,94	10,26		
Cx6F23	2	0,12	0,73	13,79	10,78		
JK-taa41	2	0,43	0,51	75,86		3,80	0,05
mCrCIR02D09	2	2,46	0,12	70,11		0,54	0,46
mCrCIR03C08	2	0,67	0,41	37,93	1,11		
mCrCIR05A05	2	3,86	0,05	75,86		3,80	0,05
mCrCIR07D05	2	1,00	0,32	26,44	4,32		
CiC0868-01	3	0,69	0,41	16,47	9,29		
CID4225	3	0,30	0,58	4,60	19,14		
CID4894	3	0,16	0,69	36,78	1,34		
CID5376	3	0,43	0,51	3,45	20,52		
CID6286	3	0,80	0,37	8,05	15,26		
CID6314	3	3,38	0,07	33,33	2,07		
CX0124	3	0,75	0,39	24,42	5,19		
MEST131	3	0,43	0,51	75,86		3,80	0,05
MEST369	3	2,67	0,10	72,41		1,49	0,22
MEST370	3	6,15	0,01	45,98	0,12		
MEST470	3	0,43	0,51	2,33	21,76		
CF-ACA01	4	0,11	0,74	9,20	14,25		
CIBE3255	4	0,89	0,35	79,31		7,19	0,01
CiC0446-01	4	2,00	0,16	79,31		7,19	0,01
CiC1757-02	4	1,35	0,25	13,95	10,67		
CiC2635-06	4	0,76	0,38	1,18	23,77		
CiC2824-01	4	0,82	0,37	8,24	15,09		
CiC2840-01	4	0,98	0,32	2,35	21,73		
CiC3740-02	4	0,08	0,78	38,82	0,92		
CiC4240-04	4	0,91	0,34	17,65	8,48		
CiC5261-01	4	0,98	0,32	2,35	21,73		
CiC6213-07	4	0,89	0,35	79,31		7,19	0,01
mCrCIR02D04b	4	1,32	0,25	78,16		5,95	0,01

mCrCIR03G05	4	1,67	0,20	82,76		11,65	0,00
MEST146	4	1,81	0,18	68,97		0,24	0,63
CiC1135-01	5	0,06	0,81	14,29	10,69		
CiC1380-05	5	0,01	0,91	1,16	23,52		
CiC2417-04	5	0,67	0,41	71,76		1,17	0,28
CiC4954-02	5	0,40	0,53	22,22	6,18		
CiC5327-03	5	0,00	1,00	6,98	16,44		
CiC5788-16	5	0,15	0,70	24,69	4,96		
CiC5842-02	5	0,13	0,72	62,79			
CID0245	5	0,05	0,83	5,75	17,88		
CID2493	5	0,33	0,56	68,97		0,24	0,63
CID5485	5	1,09	0,30	73,56		2,14	0,14
Cx6F03	5	0,36	0,55	71,26		0,95	0,33
Cx6F06	5	0,03	0,87	56,32			
mCrCIR06A12	5	0,18	0,67	74,71		2,91	0,09
MEST104	5	0,01	0,90	22,99	5,82		
CiC2128-01	6	0,33	0,56	68,24		0,11	0,74
CiC3056-02	6	2,46	0,12	70,11		0,54	0,46
CiC4033-01	6	0,76	0,38	2,41	20,39		
CiC4993-03	6	0,44	0,51	2,50	21,52		
CID5874	6	1,81	0,18	67,86		0,06	0,80
mCrCIR01C06	6	3,85	0,05	70,11		0,54	0,46
MEST123	6	6,53	0,01	65,12			
MEST132	6	2,20	0,14	36,78	1,32		
MEST191	6	0,44	0,51	4,65	19,08		
MEST322	6	1,81	0,18	68,97		0,24	0,63
MEST346	6	0,67	0,41	72,41		1,49	0,22
Ci07C07	7	2,71	0,10	4,60	19,14		
CiC1444-03	7	0,04	0,85	68,60		0,17	0,68
CiC2401-02	7	0,43	0,51	74,70		2,90	0,09
CiC3361-04	7	2,78	0,10	4,71	18,80		
CiC3674-02	7	1,00	0,32	70,93		0,82	0,37
CiC4877-04	7	1,38	0,24	69,05		0,26	0,61
CID0591	7	4,59	0,03	27,59	3,93		
mCrCIR03B07	7	1,29	0,26	27,59	3,84		
MEST107	7	0,67	0,41	72,41		1,49	0,22
MEST202	7	0,00	1,00	70,11		0,54	0,46
MEST473	7	0,62	0,43	69,41		0,34	0,56
CiC0640-03	8	0,03	0,87	54,65			
CiC1208-01	8	0,00	1,00	5,88	17,74		
CiC1749-05	8	1,29	0,26	67,06		0,01	0,93
CiC4790-02	8	1,38	0,24	67,50		0,03	0,86
CiC4853-01	8	3,60	0,06	45,35	0,16		
CMS04	8	0,33	0,56	68,97		0,24	0,63
mCrCIR01F04a	8	0,03	0,85	65,88			
mCrCIR02A09	8	0,36	0,55	71,26		0,95	0,33
mCrCIR07B05	8	2,05	0,15	32,18	2,45		
MEST502	8	0,34	0,56	16,09	9,30		
CiC0046-02	9	3,26	0,07	19,77	7,32		
CiC2768-01	9	3,07	0,08	34,52	1,80		
CiC4620-07	9	2,33	0,13	2,33	21,51		
CiC4876-07	9	2,25	0,13	81,61		10,05	0,00
CiC5087-01	9	6,00	0,01	71,08		0,88	0,35
CiC5089-06	9	2,27	0,13	47,62	0,04		
Cx6F24	9	0,53	0,47	80,46		8,56	0,00
mCrCIR07F11	9	2,71	0,10	3,45	20,52		
MEST494	9	1,45	0,23	49,43	0,00		
