# Supplementary material for: Genetic mapping of centromeres in the nine Citrus clementina chromosomes using half-tetrad analysis and recombination patterns in unreduced and haploid gametes
Source: BMC Plant Biol. 2015 Mar 8;15:80. doi: 10.1186/s12870-015-0464-y (PMC4367916; doi:10.1186/s12870-015-0464-y)
Supplement: Additional file 3: — Analysis at individual level of the origin (SDR/FDR) of clementine 2n gametes. For each of the nine LGs, we selected the marker closest to the estimated position of the centromere and computed the LOD of the probability of the individual multilocus allelic configuration under SDR/FDR hypotheses (Cuenca et al., [24]). No more than one marker in heterozygosity was observed for each individual and the LOD value supported SDR as the origin of the 2n gamete. [file 12870_2015_464_MOESM3_ESM.pdf]

Additional file 3. Analysis at individual level of the origin (SDR/FDR) of clementine 2n gametes. For each of the nine LGs, we selected the marker closest to the estimated position of the centromere and computed the LOD of the probability of the individual multilocus allelic configuration under SDR/FDR hypotheses (Cuenca et al., [24]). No more than one marker in heterozygosity was observed for each individual and the LOD value supported SDR as the origin of the 2n gamete.

**Closest marker to the centromere**	**MEST539**	**CiC6278-01**	**MEST470**	**CiC2840-01**	**CiC1380-05**	**CiC2635-06**	**Ci07C07**	**CiC1208-01**	**CiC4620-07**	
**LG**	**1**	**2**	**3**	**4**	**5**	**6**	**7**	**8**	**9**	
Centromere Position (M)	0,6066	0,5687	0,9059	0,1614	0,2312	0,064	0,9643	0,5421	0,5216	
Marker Position (M)	0,61822	0,57009	0,88762	0,16963	0,22421	0,06382	0,98019	0,58182	0,54172	
Marker distance to centromere (M)	0,01162	0,00139	0,01828	0,00823	0,00699	0,00018	0,01589	0,03972	0,02012	
**Individuals analyzed**	**Individual multilocus allelic configuration**									**LOD (SDR/FDR)**
1	HO	HO	HO	HO	HO	HO	HO	HO	HO	20,27
2	HO	HO	HE	HO		HO	HO	HO	HO	14,65
3	HO	HO	HO	HO	HO	HO	HO	HO	HO	20,27
4	HO	HO	HO	HO		HO	HO	HO	HO	18,01
5	HO	HO	HO	HO	HO	HO	HE	HO	HO	16,79
6	HO	HO	HO	HO	HO	HO	HO	HO	HO	20,27
7	HO	HO	HO	HO	HO	HO	HO	HO	HO	20,27
8	HO	HO	HO	HO	HO	HO	HO	HO	HO	20,27
9	HO	HO	HO	HO	HO	HO	HO		HO	18,82
10	HO	HO	HO	HO	HO	HO	HE	HO	HO	16,79
11	HO	HO	HO	HO	HO	HO	HE	HO	HO	16,79
12	HO	HO	HO	HO	HO	HO	HO	HO	HO	20,27
13	HO	HO	HO	HO	HO	HO	HO	HO	HO	20,27
14	HO	HO	HO	HO	HO	HO	HO	HO	HO	20,27
15	HO	HO	HO	HO	HO	HO	HO	HO	HO	20,27
16	HO	HO	HO	HO	HO	HO	HO	HO	HO	20,27
17	HO	HO	HO	HO	HO	HO	HO	HO	HO	20,27
18	HO	HO	HO	HO	HO	HO	HO	HO	HO	20,27
19	HO	HO	HO	HO	HO	HO	HO	HO	HO	20,27
20	HO	HO	HO	HO	HO	HO	HO	HO	HO	20,27
21	HO	HO	HO	HO	HO	HO	HO	HO	HO	20,27
22	HO	HO	HO	HO	HO	HO	HO	HO	HO	20,27
23	HO	HO	HO	HO	HO	HO	HO	HO	HO	20,27
24	HO	HO	HO	HO	HO	HO	HO	HO	HO	20,27
25	HO	HO	HO	HO	HO	HO	HO	HE	HO	17,66
26	HO	HO	HO	HO	HO	HO	HO	HO	HO	20,27
27	HO	HO	HO	HO	HO	HO	HO	HO	HO	20,27
28	HO	HO	HO	HO	HO	HO	HO	HO	HO	20,27
29	HO	HO	HO	HO	HO	HO	HO	HE	HO	17,66
30	HO	HO	HO	HO	HO	HO	HO	HO	HO	20,27
31	HO	HO	HO	HO	HO	HO	HO	HO	HO	20,27
32	HO	HO	HO	HO	HO	HO	HO	HO	HO	20,27
33	HO	HO	HO	HO	HO	HO	HO	HO	HO	20,27
34	HO	HO	HO		HO	HO	HO	HO	HO	18,08
35	HO	HO	HO	HO	HO	HO	HO	HO	HO	20,27
36	HO	HO		HO	HO	HO	HO	HE	HO	15,84
37	HO	HO	HO	HO	HO	HO	HO	HO	HO	20,27
38	HO	HO	HO	HO	HO	HO	HO	HO	HO	20,27
39	HO	HO	HO	HO	HO	HO	HO	HO	HO	20,27
40	HO	HO	HO	HO	HO	HO	HO	HO	HO	20,27
41	HO	HO	HO	HO	HO	HO	HO	HO	HO	20,27
42	HO	HO	HO	HO	HO	HO	HO	HO	HO	20,27
43	HO	HO	HO	HO	HO	HO	HO	HO	HO	20,27
44	HO	HO	HE	HO	HO	HO	HO	HO	HO	16,92
45	HO	HO	HO	HO	HO	HE	HO	HO	HO	12,84
46	HO	HO	HO	HO	HO	HO	HO	HO	HO	20,27
47	HO	HO	HO	HE	HO	HO	HO	HO	HO	16,19
48	HE	HO	HO	HO	HO	HO	HO	HO	HO	16,50
49	HO	HO	HO	HO	HO	HO	HO	HO	HO	20,27
50	HO	HO	HO	HO	HO	HO	HO	HO	HO	20,27
51	HO	HO	HO	HE	HO	HO	HO	HO	HO	16,19
52	HO	HO	HO	HO	HO	HO	HO	HO	HE	17,01
53	HO	HO	HO	HO	HO	HO	HO	HO	HO	20,27
54	HO	HO	HO	HO	HO	HO	HO	HO	HO	20,27
55	HO	HO	HO	HO	HO	HO	HO	HO	HO	20,27
56	HO	HO	HO	HO	HO	HO	HO	HO	HO	20,27
57	HO	HO	HO	HO	HO	HO	HO	HO	HO	20,27
58	HO	HO	HO	HO	HO	HO	HO	HO	HO	20,27
59	HO	HO	HO	HO	HO	HO	HO	HO	HO	20,27
60	HO	HO	HO	HO	HO	HO	HO	HE	HO	17,66
61	HO	HO	HO	HO	HO	HO	HO	HO	HO	20,27
62	HO	HO	HO		HO	HO	HO	HO	HO	18,08
63	HO	HO	HO	HO	HO	HO	HO	HO	HO	20,27
64	HO	HO	HO	HO	HO	HO	HO	HO	HO	20,27
65	HO	HO	HO	HO	HO	HO	HO	HO	HO	20,27
66	HO	HO		HO	HO	HO	HO	HO	HO	18,45
67	HO	HO	HO	HO	HO	HO	HO	HO	HO	20,27
68	HO	HO	HO	HO	HO	HO	HO	HO	HO	20,27
69	HO	HO	HO	HO	HO	HO	HO	HO	HO	20,27
70	HO	HO	HO	HO	HO	HO	HO	HO	HO	20,27
71	HO	HO	HO	HO	HO	HO	HO	HO	HO	20,27
72	HO	HO	HO	HO	HO	HO	HO	HO	HE	17,01
73	HO	HO	HO	HO	HO	HO	HO	HO	HO	20,27
74	HO	HO	HO	HO	HO	HO	HO	HO	HO	20,27
75	HO	HO	HO	HO	HO	HO	HO	HO	HO	20,27
76	HO	HO	HO	HO	HO	HO	HO	HO	HO	20,27
77	HO	HO	HO	HO	HO	HO	HO	HO	HO	20,27
78	HO	HO	HO	HO	HO	HO	HO	HO	HO	20,27
79	HO	HO	HO	HO	HE	HO	HO	HO	HO	16,04
80	HO	HO	HO	HO	HO	HO	HO	HO	HO	20,27
81	HO	HO	HO	HO	HO	HO	HO	HE	HO	17,66
82	HO	HO	HO	HO	HO	HO	HO	HO	HO	20,27
83	HO	HO	HO	HO	HO	HO	HO	HO	HO	20,27
84	HO	HO	HO	HO	HO	HO	HE	HO	HO	16,79
85	HO	HO	HO	HO	HO	HO	HO	HO	HO	20,27
86	HO	HO	HO	HO	HO	HO	HO	HO	HO	20,27
87	HO						HO			3,92
									**Population LOD**	**1669,28**

M. Morgans
HO. Homozygous
HE. Heterozygous
