# Supplementary material for: Genetic mapping of centromeres in the nine Citrus clementina chromosomes using half-tetrad analysis and recombination patterns in unreduced and haploid gametes
Source: BMC Plant Biol. 2015 Mar 8;15:80. doi: 10.1186/s12870-015-0464-y (PMC4367916; doi:10.1186/s12870-015-0464-y)
Supplement: Additional file 4: — Observed heterozygosity restitution (HR frequency) values for markers (squares) and theoretical functions for SDR with partial interference (x-axis indicates Kosambi’s distance) and no interference models (x-axis indicates Haldane’s distance). [file 12870_2015_464_MOESM4_ESM.pdf]

Additional file 4. Observed heterozygosity restitution (HR frequency) values for markers (squares) and theoretical functions for SDR with partial interference (x-axis indicates Kosambi's distance) and no interference models (x-axis indicates Haldane's distance).

LG 1

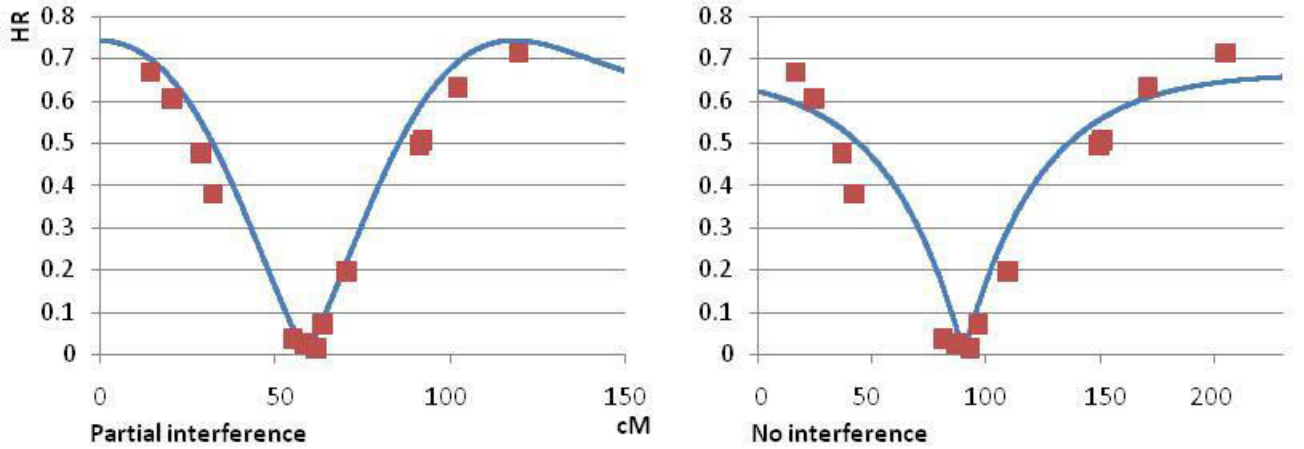

LG 2

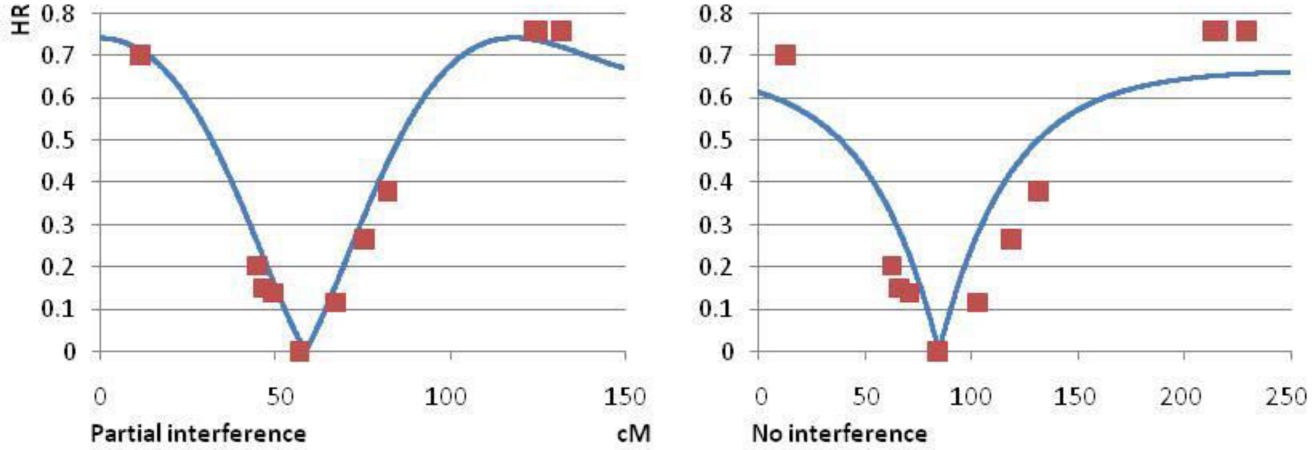

LG 3

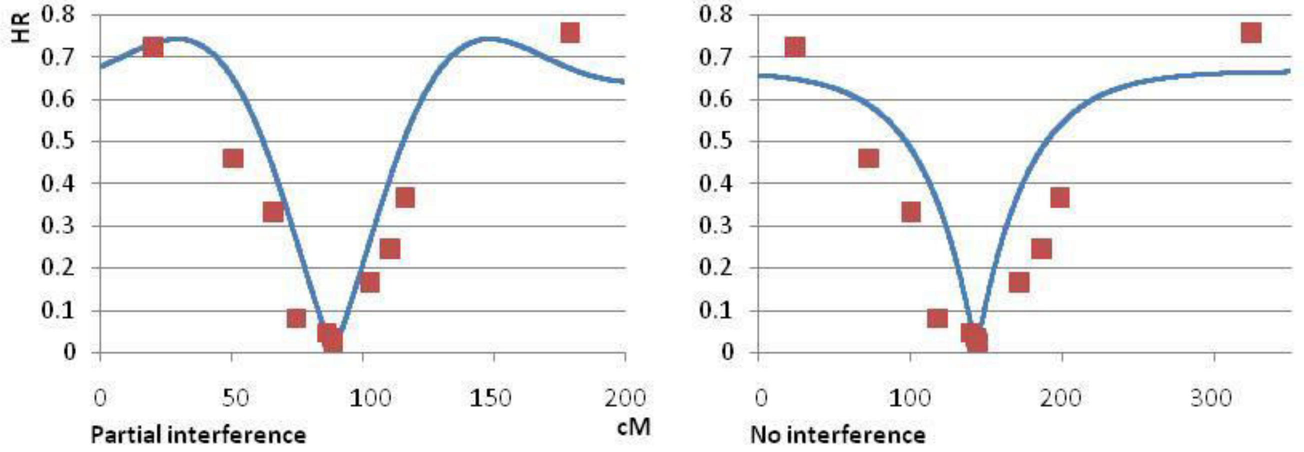

LG 4

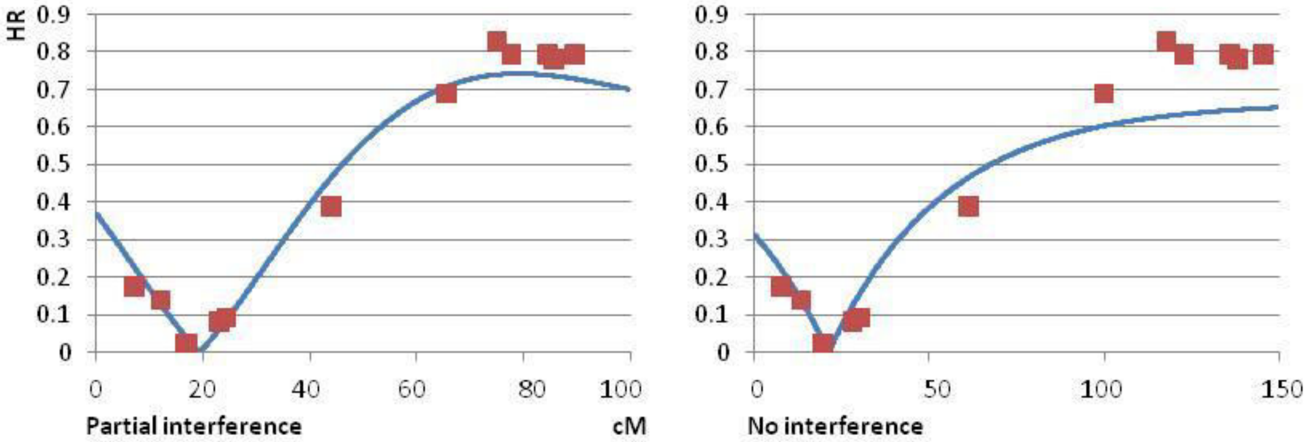
HR
0.9
0.8
0.7
0.6
0.5
0.4
0.3
0.2
0.1
0
0 20 40 60 80 100
Partial interference
cM
0.9
0.8
0.7
0.6
0.5
0.4
0.3
0.2
0.1
0
0 50 100 150
No interference

LG 5

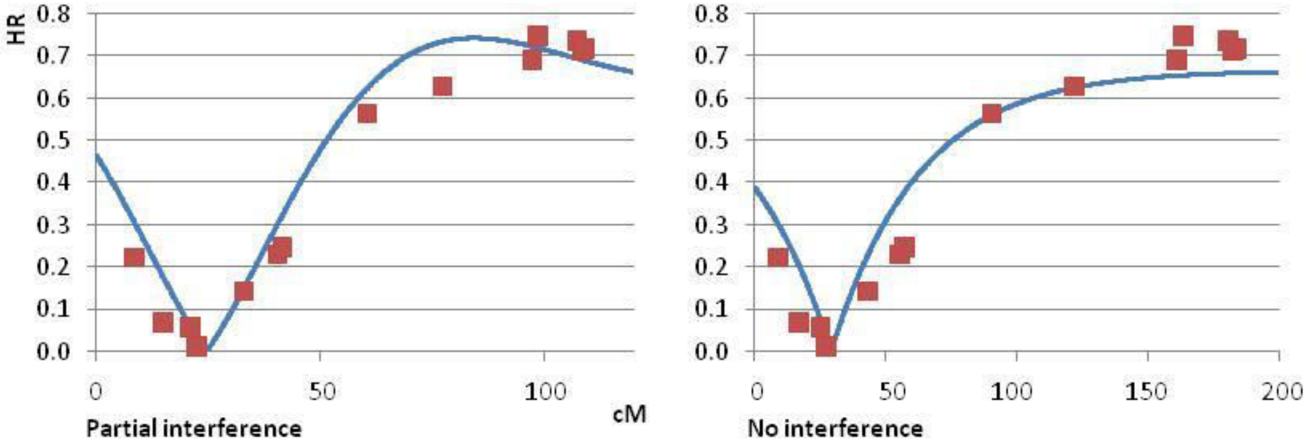
HR
0.8
0.7
0.6
0.5
0.4
0.3
0.2
0.1
0.0
0 50 100
cM
Partial interference
0.8
0.7
0.6
0.5
0.4
0.3
0.2
0.1
0.0
0 50 100 150 200
No interference

LG 6

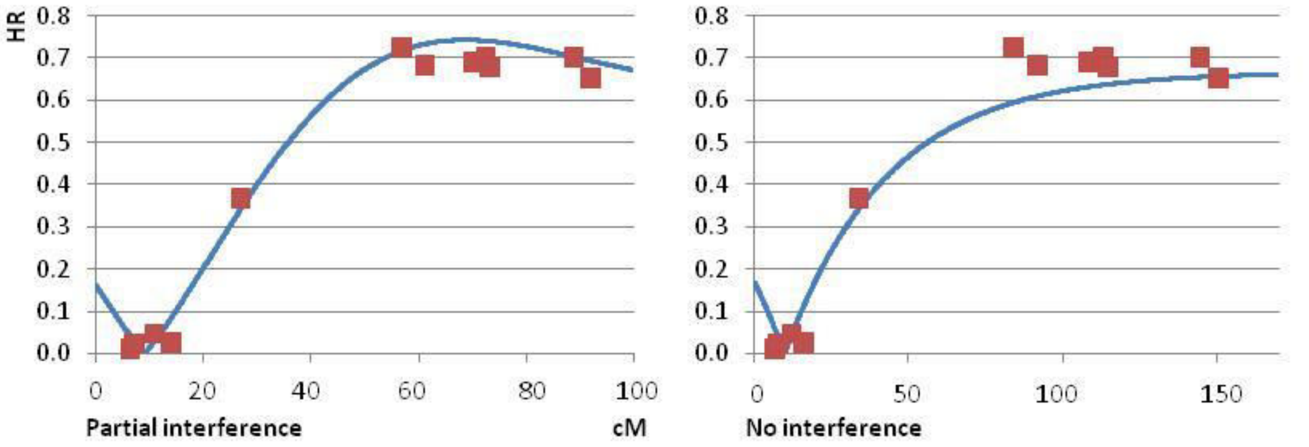
HR
0.8
0.7
0.6
0.5
0.4
0.3
0.2
0.1
0.0
0 20 40 60 80 100
Partial interference
cM
0.8
0.7
0.6
0.5
0.4
0.3
0.2
0.1
0.0
0 50 100 150
No interference

LG 7

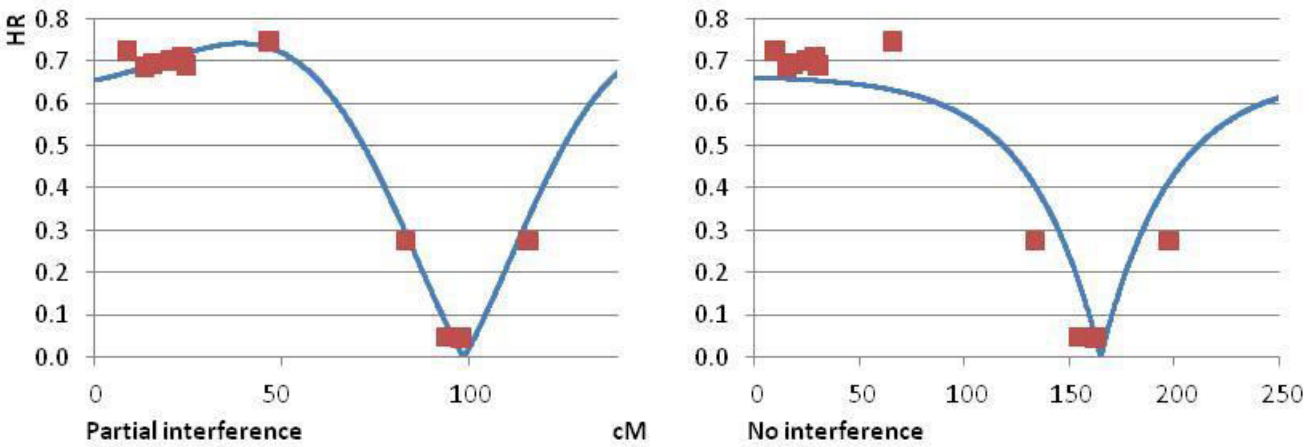
HR
0.8
0.7
0.6
0.5
0.4
0.3
0.2
0.1
0.0
0
50
100
Partial interference
cM
0
50
100
150
200
250
No interference

LG 8

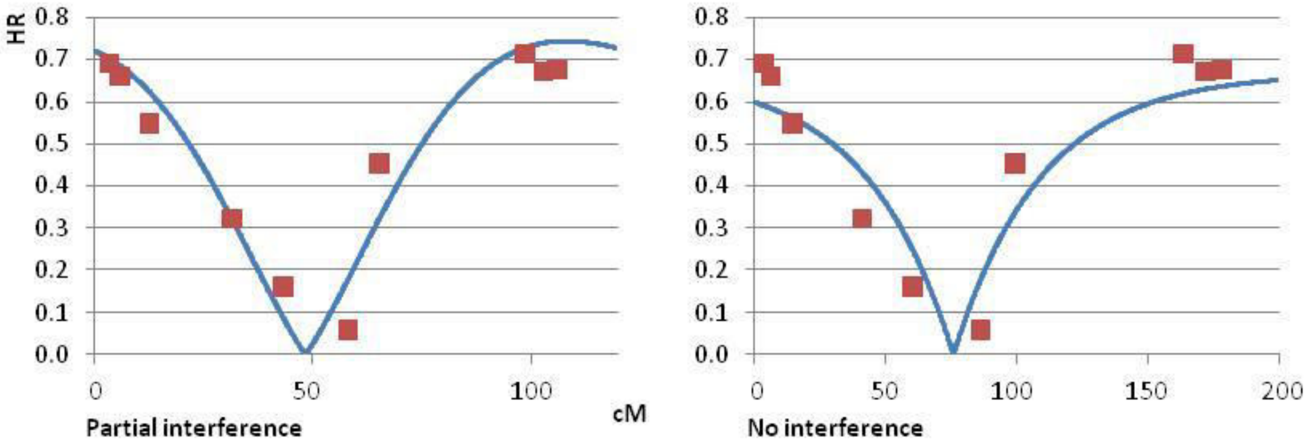
HR
0.8
0.7
0.6
0.5
0.4
0.3
0.2
0.1
0.0
0
50
100
Partial interference
cM
0
50
100
150
200
No interference

LG 9

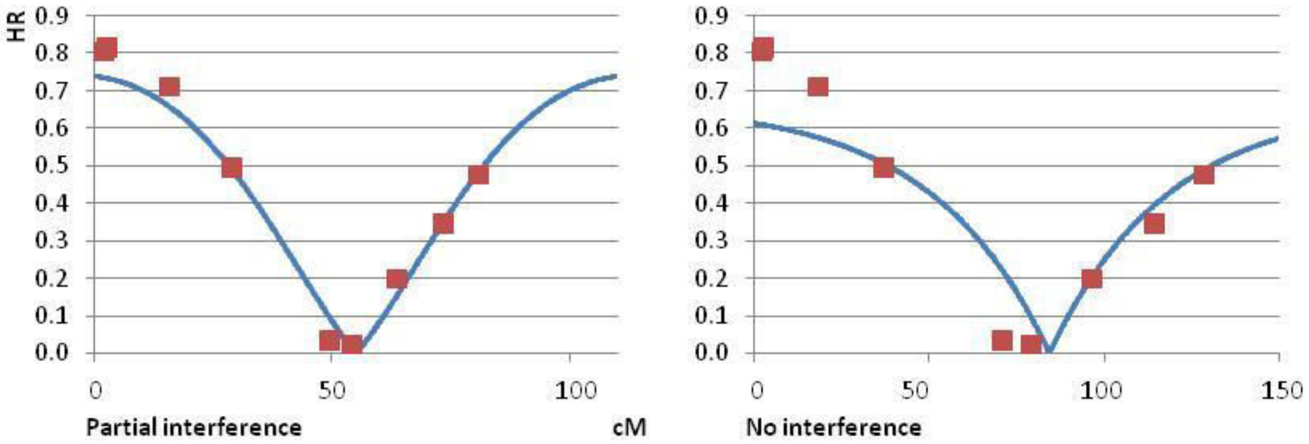
HR
0.9
0.8
0.7
0.6
0.5
0.4
0.3
0.2
0.1
0.0
0
50
100
Partial interference
cM
0
50
100
150
No interference
