# Supplementary material for: Genetic mapping of centromeres in the nine Citrus clementina chromosomes using half-tetrad analysis and recombination patterns in unreduced and haploid gametes
Source: BMC Plant Biol. 2015 Mar 8;15:80. doi: 10.1186/s12870-015-0464-y (PMC4367916; doi:10.1186/s12870-015-0464-y)
Supplement: Additional file 5: — Heterozygous and homozygous profiles for 2n gametes analysed by HTA using four markers flanking the position of the centromere in each LG. N: Number of triploid hybrids for each profile; M: Name of the molecular marker; HR%: Heterozygosity restitution value; HE: Heterozygous; HO: Homozygous. [file 12870_2015_464_MOESM5_ESM.pdf]

Additional file 5. Heterozygous and homozygous profiles for 2n gametes analysed by HTA using four markers flanking the position of the centromere in each LG. N: Number of triploid hybrids for each profile; M: Name of the molecular marker; HR%: Heterozygosity restitution value; HE: Heterozygous; HO: Homozygous.

**LG1**	CID0806	CIBE5720	MEST539	CiC4581-01
N	M1	M2	M3	M4
0	HE	HE	HE	HE
0	HE	HE	HE	HO
0	HE	HE	HO	HE
0	HE	HO	HE	HE
0	HO	HE	HE	HE
1	HE	HE	HO	HO
0	HE	HO	HE	HO
0	HE	HO	HO	HE
1	HO	HO	HE	HE
0	HO	HE	HO	HE
0	HO	HE	HE	HO
5	HO	HO	HO	HE
0	HO	HO	HE	HO
1	HO	HE	HO	HO
2	HE	HO	HO	HO
76	HO	HO	HO	HO
86				
HR%	3,45	2,30	1,15	6,98

**LG2**	Cx2004	Cx6F23	CiC6278-01	CiC3440-07
N	M1	M2	M3	M4
0	HE	HE	HE	HE
0	HE	HE	HE	HO
1	HE	HE	HO	HE
0	HE	HO	HE	HE
0	HO	HE	HE	HE
11	HE	HE	HO	HO
0	HE	HO	HE	HO
0	HE	HO	HO	HE
0	HO	HO	HE	HE
0	HO	HE	HO	HE
0	HO	HE	HE	HO
9	HO	HO	HO	HE
0	HO	HO	HE	HO
0	HO	HE	HO	HO
1	HE	HO	HO	HO
64	HO	HO	HO	HO
86				
	14,94	13,79	0,00	11,63

**LG3**	CID4225	CID5376	MEST470	CiC0868-01
N	M1	M2	M3	M4
0	HE	HE	HE	HE
0	HE	HE	HE	HO
0	HE	HE	HO	HE
0	HE	HO	HE	HE
0	HO	HE	HE	HE
1	HE	HE	HO	HO
1	HE	HO	HE	HO
0	HE	HO	HO	HE
0	HO	HO	HE	HE
0	HO	HE	HO	HE
1	HO	HE	HE	HO
13	HO	HO	HO	HE
0	HO	HO	HE	HO
1	HO	HE	HO	HO
2	HE	HO	HO	HO
65	HO	HO	HO	HO
84				
	4,60	3,45	2,33	16,47

**LG4**	CiC1757-02	CiC2840-01	CiC2824-01	CF-ACA01
N	M1	M2	M3	M4
2	HE	HE	HE	HE
0	HE	HE	HE	HO
0	HE	HE	HO	HE
0	HE	HO	HE	HE
0	HO	HE	HE	HE
0	HE	HE	HO	HO
0	HE	HO	HE	HO
1	HE	HO	HO	HE
3	HO	HO	HE	HE
0	HO	HE	HO	HE
0	HO	HE	HE	HO
2	HO	HO	HO	HE
2	HO	HO	HE	HO
0	HO	HE	HO	HO
8	HE	HO	HO	HO
66	HO	HO	HO	HO
84				
HR%	13,95	2,35	8,24	9,20

**LG5**	CiC5327-03	CID0245	CiC1380-05	CiC1135-01
N	M1	M2	M3	M4
1	HE	HE	HE	HE
0	HE	HE	HE	HO
0	HE	HE	HO	HE
0	HE	HO	HE	HE
0	HO	HE	HE	HE
0	HE	HE	HO	HO
0	HE	HO	HE	HO
0	HE	HO	HO	HE
0	HO	HO	HE	HE
0	HO	HE	HO	HE
0	HO	HE	HE	HO
11	HO	HO	HO	HE
0	HO	HO	HE	HO
3	HO	HE	HO	HO
5	HE	HO	HO	HO
64	HO	HO	HO	HO
84				
H	6,98	5,75	1,16	14,29

**LG6**	CiC2635-06	CiC4033-01	MEST191	CiC4993-03
	M1	M2	M3	M4
0	HE	HE	HE	HE
0	HE	HE	HE	HO
0	HE	HE	HO	HE
0	HE	HO	HE	HE
1	HO	HE	HE	HE
0	HE	HE	HO	HO
0	HE	HO	HE	HO
0	HE	HO	HO	HE
1	HO	HO	HE	HE
0	HO	HE	HO	HE
0	HO	HE	HE	HO
1	HO	HO	HO	HE
2	HO	HO	HE	HO
0	HO	HE	HO	HO
0	HE	HO	HO	HO
79	HO	HO	HO	HO
84				
H	1,16	2,33	4,65	3,53

**LG7**	Ci03B07	CiC3361-04	Ci07C07	CID0591
N	M1	M2	M3	M4
2	HE	HE	HE	HE
0	HE	HE	HE	HO
0	HE	HE	HO	HE
1	HE	HO	HE	HE
0	HO	HE	HE	HE
2	HE	HE	HO	HO
0	HE	HO	HE	HO
3	HE	HO	HO	HE
0	HO	HO	HE	HE
0	HO	HE	HO	HE
0	HO	HE	HE	HO
16	HO	HO	HO	HE
0	HO	HO	HE	HO
0	HO	HE	HO	HO
15	HE	HO	HO	HO
46	HO	HO	HO	HO
85				
HR%	27,59	4,71	4,60	27,59

**LG8**	Ci07B05	MEST502	CiC1208-01	CiC4853-01
N	M1	M2	M3	M4
1	HE	HE	HE	HE
0	HE	HE	HE	HO
3	HE	HE	HO	HE
2	HE	HO	HE	HE
0	HO	HE	HE	HE
4	HE	HE	HO	HO
0	HE	HO	HE	HO
11	HE	HO	HO	HE
2	HO	HO	HE	HE
0	HO	HE	HO	HE
0	HO	HE	HE	HO
19	HO	HO	HO	HE
0	HO	HO	HE	HO
7	HO	HE	HO	HO
7	HE	HO	HO	HO
29	HO	HO	HO	HO
85				
H	32,18	16,09	5,88	45,35

**LG9**	MEST494	Ci07F11	CiC4620-07	CiC0046-02
N	M1	M2	M3	M4
1	HE	HE	HE	HE
0	HE	HE	HE	HO
1	HE	HE	HO	HE
1	HE	HO	HE	HE
0	HO	HE	HE	HE
1	HE	HE	HO	HO
0	HE	HO	HE	HO
6	HE	HO	HO	HE
0	HO	HO	HE	HE
0	HO	HE	HO	HE
0	HO	HE	HE	HO
8	HO	HO	HO	HE
0	HO	HO	HE	HO
0	HO	HE	HO	HO
32	HE	HO	HO	HO
36	HO	HO	HO	HO
86				
H	49,43	3,45	2,33	19,77
