# Supplementary material for: Genetic mapping of centromeres in the nine Citrus clementina chromosomes using half-tetrad analysis and recombination patterns in unreduced and haploid gametes
Source: BMC Plant Biol. 2015 Mar 8;15:80. doi: 10.1186/s12870-015-0464-y (PMC4367916; doi:10.1186/s12870-015-0464-y)

Additional file 6. Distribution of the number of crossovers observed in 2n gametes on each arm of each LGs.

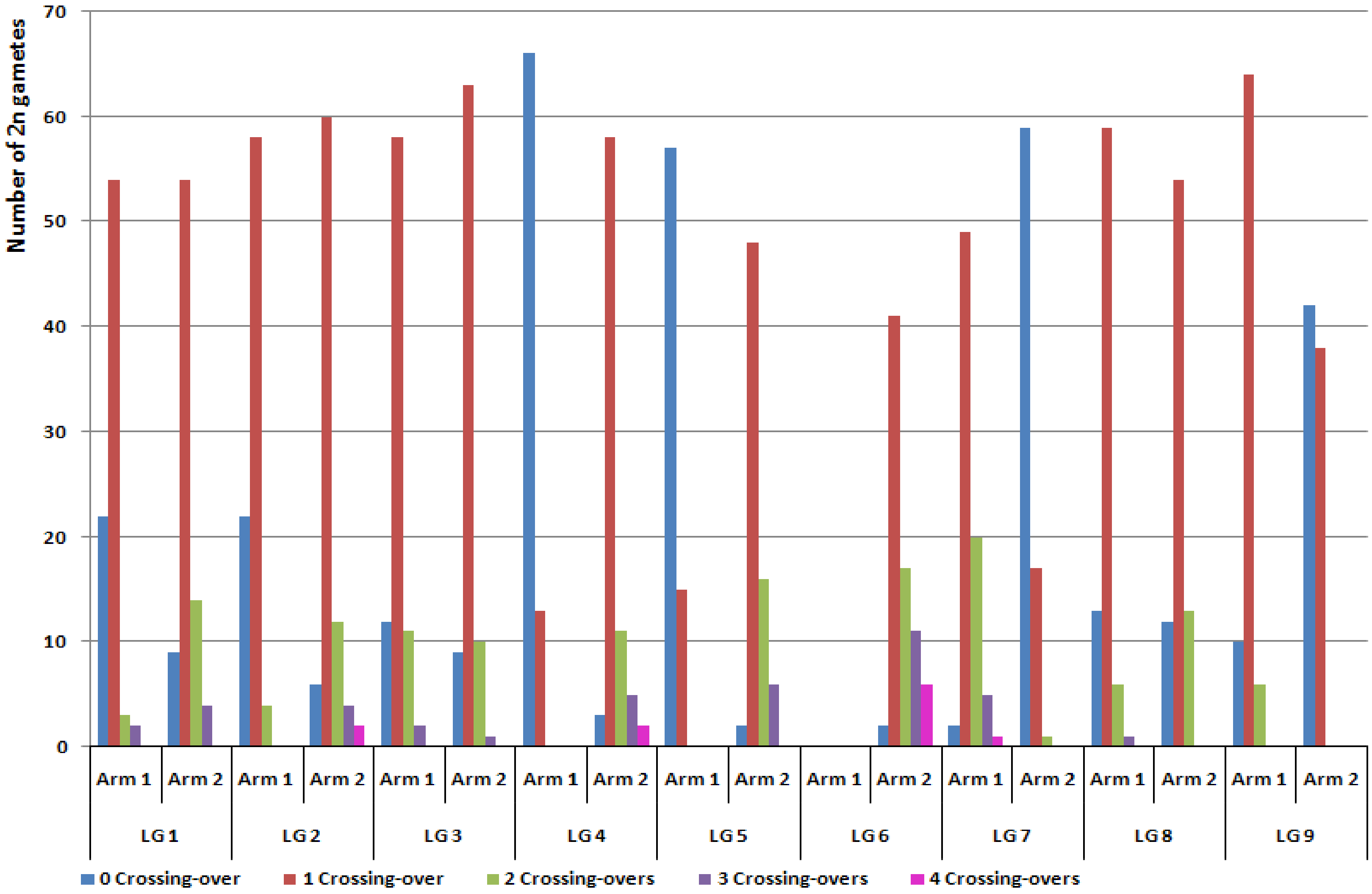

Supplement: Additional file 6: — Distribution of the number of crossovers observed in 2n gametes on each arm of each LGs. [file 12870_2015_464_MOESM6_ESM.pdf]
