# Supplementary material for: Genetic mapping of centromeres in the nine Citrus clementina chromosomes using half-tetrad analysis and recombination patterns in unreduced and haploid gametes
Source: BMC Plant Biol. 2015 Mar 8;15:80. doi: 10.1186/s12870-015-0464-y (PMC4367916; doi:10.1186/s12870-015-0464-y)
Supplement: Additional file 7: — Analysis of crossover interference in clementine. Heterozygous (HE, ab) and homozygous (HO, aa and bb) profiles of 2n gametes analysed using 11 markers. The location of the centromere on LG 3 was estimated using HTA. Numbers in grey are the number of 2n gametes with different crossover events on one arm and between arms (0, 1, 2, 3 and 4). Numbers in bold correspond to percentages of crossovers observed in each chromosome arm. Potential interference between arms was tested for each chromosome using χ2-analysis based on the contingency table of the number of crossovers (grey numbers) in each chromosome arm. [file 12870_2015_464_MOESM7_ESM.pdf]

Genotype	M1	M2	M3	M4	M5	M6	M7	*CENTROMERE*	M8	M9	M10	M11
1	ab	ab	ab	ab	ab	ab	aa	*HO*	aa	aa	ab	aa
2	ab	aa	aa	aa	aa	ab	ab	*HO*	aa	aa	aa	ab
3	ab	ab	ab	aa	aa	aa	aa	*HO*	aa	aa	aa	ab
4	ab	bb	bb	bb	bb	bb	bb	*HO*	bb	bb	bb	ab
5	aa	aa	aa	aa	aa	aa	aa	*HO*	ab	ab	ab	ab
6	ab	ab	aa	ab	aa	ab	aa	*HO*	aa	aa	ab	ab
7	ab	aa	aa	aa	aa	aa	aa	*HO*	aa	aa	aa	ab
8	ab	bb	bb	bb	bb	bb	bb	*HO*	bb	bb	bb	ab
9	ab	bb	bb	bb	bb	bb	bb	*HO*	ab	ab	ab	ab
10	bb	bb	bb	bb	bb	bb	bb	*HO*	ab	ab	ab	ab
11	ab	ab	ab	aa	aa	aa	aa	*HO*	ab	ab	ab	aa
12	aa	ab	ab	bb	bb	bb	bb	*HO*	bb	bb	bb	aa
13	ab	ab	ab	aa	aa	aa	aa	*HO*	aa	aa	aa	ab
14	ab	ab	ab	bb	bb	bb	bb	*HO*	bb	bb	bb	ab
15	ab	bb	bb	bb	bb	bb	bb	*HO*	ab	ab	ab	ab
16	ab	ab	bb	bb	bb	bb	bb	*HO*	ab	ab	ab	bb
17	ab	aa	aa	aa	aa	aa	aa	*HO*	aa	aa	aa	ab
18	ab	ab	ab	bb	bb	bb	bb	*HO*	bb	bb	bb	ab
19	ab	aa	aa	aa	aa	aa	aa	*HO*	aa	aa	aa	aa
20	aa	aa	aa	aa	bb	bb	bb	*HO*	bb	bb	bb	ab
21	ab	ab	ab	bb	bb	bb	bb	*HO*	bb	bb	ab	ab
22	ab	aa	aa	aa	aa	aa	aa	*HO*	aa	aa	aa	aa
23	ab	aa	aa	aa	aa	aa	aa	*HO*	aa	aa	ab	ab
24	ab	aa	aa	ab	bb	bb	bb	*HO*	ab	ab	ab	ab
25	ab	ab	aa	aa	aa	aa	aa	*HO*	aa	aa	aa	ab
26	ab	ab	ab	bb	bb	bb	bb	*HO*	ab	ab	ab	bb
27	aa	aa	aa	aa	aa	aa	aa	*HO*	aa	aa	aa	bb
28	ab	aa	aa	aa	aa	aa	aa	*HO*	ab	ab	ab	ab
29	bb	ab	ab	aa	aa	aa	aa	*HO*	aa	aa	aa	ab
30	ab	ab	ab	aa	aa	aa	aa	*HO*	aa	ab	ab	ab
31	ab	ab	ab	aa	aa	aa	aa	*HO*	ab	ab	ab	bb
32	ab	bb	bb	bb	bb	bb	bb	*HO*	bb	bb	bb	ab
33	aa	aa	aa	aa	aa	aa	aa	*HO*	bb	bb	bb	bb
34	aa	aa	aa	aa	aa	aa	aa	*HO*	bb	bb	bb	bb
35	ab	ab	bb	bb	bb	bb	bb	*HO*	bb	bb	bb	ab
36	aa	ab	ab	bb	bb	bb	bb	*HO*		bb	bb	ab
37	ab	aa	aa	aa	aa	aa	aa	*HO*	aa	aa	aa	aa
38	ab	ab	ab	bb	bb	bb	bb	*HO*	bb	bb	bb	ab
39	ab	aa	aa	aa	aa	aa	aa	*HO*	aa	aa	aa	ab
40	ab	aa	aa	aa	aa	aa	aa	*HO*	aa	aa	aa	ab
41	bb	bb	bb	bb	bb	bb	bb	*HO*	bb	bb	bb	bb
42	ab	ab	ab	bb	bb	bb	bb	*HO*	bb	bb	bb	ab
43	bb	bb	bb	bb	bb	bb	bb	*HO*	bb	bb	bb	ab
44	ab	ab	ab	ab	ab	aa	ab	*HO*	aa	aa	aa	ab
45	ab	ab	bb	bb	bb	bb	bb	*HO*	bb	bb	bb	ab
46	aa	aa	aa	aa	aa	aa	aa	*HO*	aa	ab	ab	ab
47	ab	aa	aa	aa	aa	aa	aa	*HO*	aa	aa	ab	bb
48	aa	ab	ab	bb	bb	bb	bb	*HO*	bb	ab	ab	ab
49	ab	ab	bb	bb	bb	bb	bb	*HO*	bb	bb	bb	ab
50	ab	ab	bb	bb	bb	bb	bb	*HO*	bb	bb	bb	ab
51	ab	aa	aa	aa	aa	aa	aa	*HO*	aa	aa	aa	bb
52	aa	ab	ab	bb	bb	bb	bb	*HO*	bb	bb	ab	ab
53	ab	ab	ab	aa	aa	aa	aa	*HO*	ab	ab	aa	ab
54	ab	aa	aa	aa	aa	aa	aa	*HO*	aa	aa	aa	ab
55	ab	bb	bb	bb	bb	bb	bb	*HO*	bb	ab	ab	bb
56	ab	ab	ab	ab	ab	aa	aa	*HO*	aa	aa	ab	ab
57	ab	aa	aa	aa	aa	aa	aa	*HO*	aa	aa	aa	ab
58	bb	bb	bb	bb	bb	bb	bb	*HO*	bb	ab	ab	ab
59	aa	aa	aa	aa	aa	aa	aa	*HO*	aa	aa	ab	ab
60	ab	ab	bb	bb	bb	bb	bb	*HO*	bb	bb	bb	ab
61	aa	ab	aa	aa	aa	aa	aa	*HO*	aa	aa	aa	ab
62	ab	aa	aa	aa	aa	aa	aa	*HO*	ab	ab	ab	ab
63	ab	bb	bb	bb	bb	bb	bb	*HO*	bb	bb	bb	ab
64	ab	aa	aa	aa	aa	aa	aa	*HO*	aa	aa	aa	ab
65	aa	aa	aa	aa	aa	aa	aa	*HO*	aa	aa	aa	ab
66	ab	ab	aa	aa	aa	aa		*HO*	ab		ab	aa
67	ab	bb	bb	bb	bb	bb	bb	*HO*	bb	bb	bb	ab
68	ab	ab	aa	aa	aa	aa	aa	*HO*	aa	aa	aa	ab
69	aa	aa	ab	ab	bb	bb	bb	*HO*	bb	ab	ab	ab
70	bb	bb	bb	bb	bb	bb	bb	*HO*	ab	ab	ab	ab
71	ab	ab	aa	aa	aa	aa	aa	*HO*	aa	aa	aa	ab
72	ab	ab	aa	aa	aa	aa	aa	*HO*	aa	aa	ab	ab
73	aa	aa	bb	bb	bb	bb	bb	*HO*	bb	bb	bb	bb
74	ab	ab	ab	bb	bb	bb	bb	*HO*	bb	bb	bb	bb
75	ab	ab	ab	aa	aa	aa	aa	*HO*	aa	aa	aa	ab
76	ab	aa	aa	aa	aa	aa	aa	*HO*	aa	aa	ab	ab
77	aa	aa	ab	aa	aa	aa	aa	*HO*	aa	aa	aa	aa
78	ab	ab	ab	bb	bb	bb	bb	*HO*	bb	bb	bb	ab
79	ab	aa	aa	aa	aa	aa	aa	*HO*	aa	aa	aa	ab
80	ab	ab	ab	aa	aa	aa	aa	*HO*	aa	ab	ab	ab
81	ab	ab	ab	ab	ab	aa	aa	*HO*	aa	aa	aa	ab
82	ab	ab	bb	bb	bb	bb	bb	*HO*	bb	bb	bb	ab
83	ab	bb	bb	bb	bb	bb	bb	*HO*	bb	bb	bb	ab
84	ab	aa	aa	aa	aa	aa	aa	*HO*	aa	aa	aa	ab
85	bb	ab	ab	bb	bb	bb	bb	*HO*	bb	bb	ab	ab
86	ab	aa	aa	aa	aa	aa	aa	*HO*	aa	aa	aa	ab
87	bb	bb	ab	bb	bb	bb	bb	*HO*		ab	ab	aa

**Nº CO on LG 3**		**Arm 1**					
		**0**	**1**	**2**	**3**	**4**	
**Arm 2**	**0**	3	4	2	0	0	**10.8**
	**1**	8	45	8	2	0	**75.9**
	**2**	1	8	1	0	0	**12.0**
	**3**	0	1	0	0	0	**1.2**
	**4**	0	0	0	0	0	**0.0**
		**14.5**	**69.9**	**13.3**	**2.41**	**0.0**	

Chi squared analysis of LG 3 based on contingency table

Chi-squared (Observed value)	**5.55**
Chi-squared (Critical value)	**21.03**
Freedom degree	**12**
p-value	**0.94**
Significance level	**0.05**

Additional file 7. Analysis of crossover interference in clementine. Heterozygous (HE, ab) and homozygous (HO, aa and bb) profiles of 2n gametes analysed using 11 markers. The location of the centromere on LG 3 was estimated using HTA. Numbers in grey are the number of 2n gametes with different crossover events on one arm and between arms (0, 1, 2, 3 and 4). Numbers in bold correspond to percentages of crossovers observed in each chromosome arm. Potential interference between arms was tested for each chromosome using χ2-analysis based on the contingency table of the number of crossovers (grey numbers) in each chromosome arm.
