# Supplementary material for: Genetic mapping of centromeres in the nine Citrus clementina chromosomes using half-tetrad analysis and recombination patterns in unreduced and haploid gametes
Source: BMC Plant Biol. 2015 Mar 8;15:80. doi: 10.1186/s12870-015-0464-y (PMC4367916; doi:10.1186/s12870-015-0464-y)
Supplement: Additional file 8: — Variation in recombination rate along the different clementine chromosomes (Chr). The x-axis shows the physical position in megabases along each chromosome and the y-axis represents the ratio of genetic distance to physical distance (cM/Mb). The bars beneath the x-axis indicate the approximate locations of the centromeres (CI). * These data have been calculated from up and down intervals (no marker on the genetic map on the considered genomic segment). [file 12870_2015_464_MOESM8_ESM.pdf]

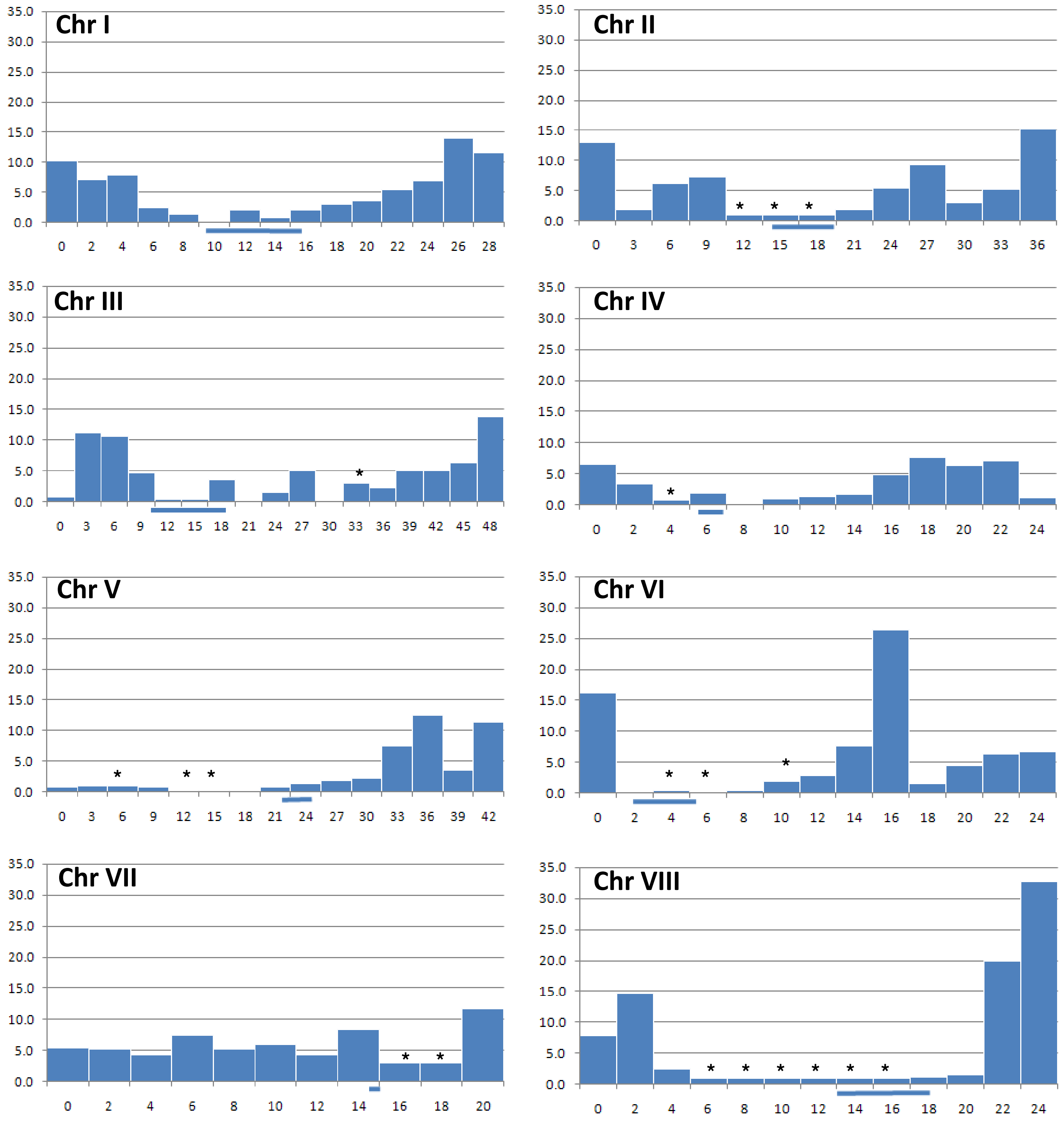

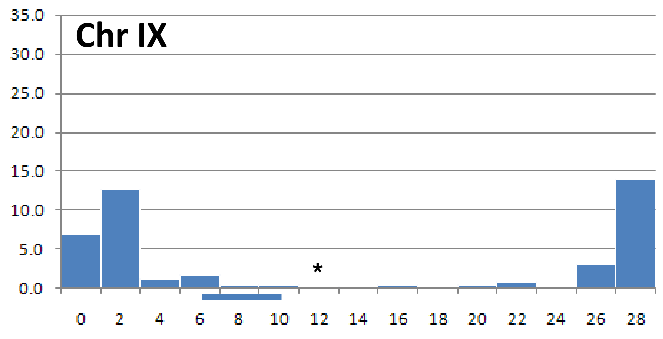

Additional file 8. Variation in recombination rate along the different clementine chromosomes (Chr). The x-axis shows the physical position in megabases along each chromosome and the y-axis represents the ratio of genetic distance to physical distance (cM/Mb). The bars beneath the x-axis indicate the approximate locations of the centromeres (CI).
*** These data have been calculated from up and down intervals (no marker on the genetic map on the considered genomic segment).**
