# Supplementary material for: Genetic mapping of centromeres in the nine Citrus clementina chromosomes using half-tetrad analysis and recombination patterns in unreduced and haploid gametes
Source: BMC Plant Biol. 2015 Mar 8;15:80. doi: 10.1186/s12870-015-0464-y (PMC4367916; doi:10.1186/s12870-015-0464-y)

Additional file 9. Relationship between the physical location (x-axis), the genetic distance between centromere and markers on the clementine genetic map (black dots) and the proportion of gene sequences (blue bars) along all chromosomes (Chr). Bars beneath the x-axis indicate the approximate locations of the centromeres location (Cl).

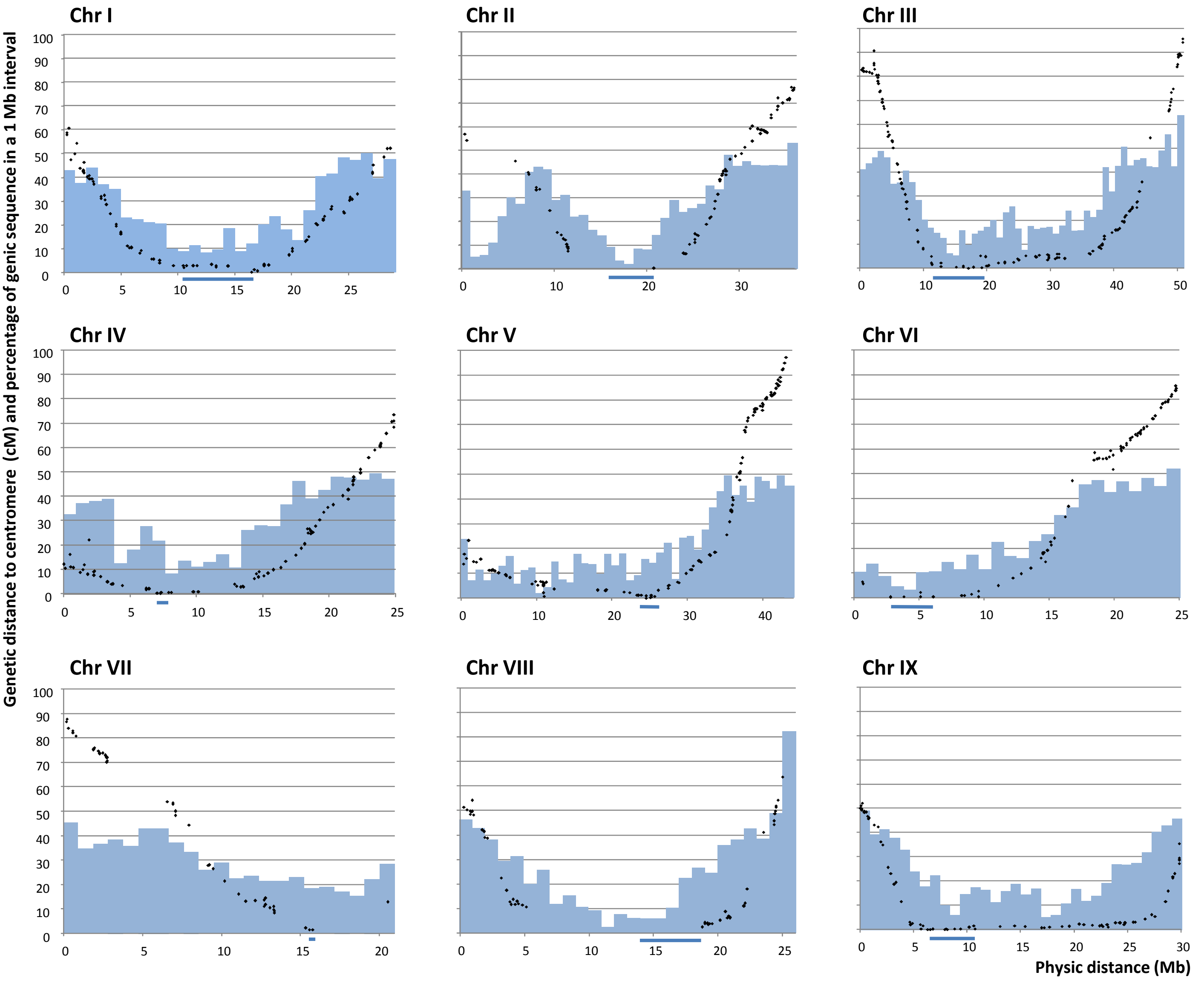

Supplement: Additional file 9: — Relationship between the physical location (x-axis), the genetic distance between centromere and markers on the clementine genetic map (black dots) and the proportion of gene sequences (blue bars) along all chromosomes. Bars beneath the x-axis indicate the approximate locations of the centromeres location (CI). [file 12870_2015_464_MOESM9_ESM.pdf]
